# Prediction of polyspecificity from antibody sequence data by machine learning

**DOI:** 10.3389/fbinf.2023.1286883

**Published:** 2024-04-08

**Authors:** Szabolcs Éliás, Clemens Wrzodek, Charlotte M. Deane, Alain C. Tissot, Stefan Klostermann, Francesca Ros

**Affiliations:** ^1^ Roche Pharma Research and Early Development Informatics, Roche Innovation Center Munich, Penzberg, Germany; ^2^ Oxford Protein Informatics Group, Department of Statistics, University of Oxford, Oxford, United Kingdom; ^3^ Roche Pharmaceutical Research and Early Development, Large Molecule Research, Roche Innovation Center Munich, Penzberg, Germany

**Keywords:** neural network, immunoglobulin, immune repertoire, polyspecificity, antibody, therapeutic antibodies, deep learning, machine learning

## Abstract

Antibodies are generated with great diversity in nature resulting in a set of molecules, each optimized to bind a specific target. Taking advantage of their diversity and specificity, antibodies make up for a large part of recently developed biologic drugs. For therapeutic use antibodies need to fulfill several criteria to be safe and efficient. Polyspecific antibodies can bind structurally unrelated molecules in addition to their main target, which can lead to side effects and decreased efficacy in a therapeutic setting, for example via reduction of effective drug levels. Therefore, we created a neural-network-based model to predict polyspecificity of antibodies using the heavy chain variable region sequence as input. We devised a strategy for enriching antibodies from an immunization campaign either for antigen-specific or polyspecific binding properties, followed by generation of a large sequencing data set for training and cross-validation of the model. We identified important physico-chemical features influencing polyspecificity by investigating the behaviour of this model. This work is a machine-learning-based approach to polyspecificity prediction and, besides increasing our understanding of polyspecificity, it might contribute to therapeutic antibody development.

## 1 Introduction

Antibodies produced by B lymphocytes are a crucial part of the adaptive immune system. They are large proteins recognizing certain structures in their cognate antigen, and specific antibodies are generated during a germinal center reaction in secondary lymphoid organs [reviewed in (Victora and Nussenzweig, 2012)]. Antibody binding tags a pathogenic structure, ultimately leading to its neutralization and/or elimination by a complex interplay of several immune cells and pathways.

Genes encoding antibodies contain variable segments which through gene segment rearrangements, iterative somatic mutations and subsequent selections of antigen-binding antibodies enable the creation of a large variety of antibodies recognizing virtually any potentially hazardous infectious invader from a limited set of genes. Due to this versatility, antibodies specific against almost any desired antigen can be created and thus have been developed as research tools and for therapeutic purposes. Antibodies or B cell receptors, as well as T cell receptors (TCRs), are adaptive immune receptors (AIRs), and nature must balance to have a diverse repertoire enabling any unknown pathogen to be recognized, yet maintaining sufficient specificity (Rappazzo et al., 2023). Understanding, describing and experimentally studying these features including undesired features such as polyreactivity, also known as polyspecificity, are a key challenge to the field (Rappazzo et al., 2023), especially given the growing potential of antibodies and TCRs as drugs or in cell therapies.

In recent years, there is an increasing number of clinically approved therapeutic antibodies used to treat different diseases from cancer to autoimmune disease, and more antibodies as well as multispecific antibody formats are expected to come in the future (Elgundi et al., 2016). Antibodies usable as drugs must fulfill certain favorable biophysical properties such as high solubility and stability paired with low potential for aggregation, and several unfavorable properties have been associated with their hydrophobicity (Tsai and Nussinov, 1997; Chennamsetty et al., 2009).

Binding to the target antigen is a crucial feature of an antibody, however, this binding has to be specific, and unspecific binding to other antigens such as self-antigens could even contribute to autoimmune diseases. So-called polyspecific antibodies bind to a variety of different structurally unrelated antigens and due to potential off-target effects are a concern for therapeutic antibody development [reviewed in (Dimitrov et al., 2013)]. Nevertheless, there is incomplete knowledge about which properties make an antibody polyspecific, and prediction of such attributes would be highly desirable to improve the design and development of therapeutic antibodies.

While polyspecific antibodies may fulfill important roles in providing broadly neutralizing protective function against pathogens (Ochsenbein et al., 1999; Zhou et al., 2007; Planchais et al., 2019), they have also been suggested to be related to unwanted autoreactive antibodies in autoimmune diseases such as in systemic lupus erythematosus (Mietzner et al., 2008; Zhang et al., 2009). An important source of polyreactive antibodies *in vivo* are so-called B1 cells with innate-like properties (Prieto and Felippe, 2017).

Opposed to polyspecific antibodies present in the pre-immune repertoire, immunization and adaptive response to an antigen lead to an increase of specificity and affinity over time due to affinity maturation, that is the process of somatic hypermutation followed by selection of clones producing high-affinity antibodies. Accordingly, polyreactive antibodies are often encoded by germline genes or genes with limited somatic hypermutation (Baccala et al., 1989; Sequeira et al., 1992). Complementarity-determining regions (CDRs) are the most variable part of an antibody and crucially determine the antigen binding site. Especially the CDR3 loop in the heavy chain (Deng and Notkins, 2000), together with some germline-like segments, plays the main role in determining whether an antibody displays polyreactivity (Ichiyoshi and Casali, 1994). The immature light chain has also been shown to contribute to polyspecificity (Witsch et al., 2006). Furthermore, computational design has revealed that antibodies designed to be polyspecific are likely to be similar to germline repertoires, while those designed to be specific are likely to be similar to affinity-matured antibodies (Willis et al., 2013).

These observations support the concept that antibody maturation leads to increased specificity (Oppezzo et al., 2004). Indeed, early human B cell precursors were described to have more polyreactive clones (Wardemann et al., 2003). Different experimental screening assays have been developed to determine polyspecificity such as binding assessment to sets of structurally divergent antigens (for example, baculovirus particle, DNA, flagellin, albumin, LPS) by ELISA- or flow-cytometry-based assays (Wardemann et al., 2003; Xu et al., 2013; Jain et al., 2017).

A recent thorough study on human antibodies across developmental stages of B cells has used such polyspecificity assays along with methods to test for other “undesirable” properties of antibodies, and has determined that affinity matured human antibodies show reduced polyreactivity and hydrophobicity as compared to antibodies derived from naïve B cells (Shehata et al., 2019). Nevertheless, this study remains limited to a few hundred antibodies from human subjects in the absence of interventional immunization with a model antigen.

To overcome these limitations, we designed experiments to immunize transgenic rabbits with different model antigens followed by next-generation sequencing (NGS) analysis of the pre-immune and immune antibody repertoire. We combined this design with binding assays to the model antigen or a mixture of typical antigens for polyspecific antibodies, followed by machine-learning-based modeling using the heavy chain variable region sequences. To enable this, we took advantage of transgenic rabbits displaying a common immunoglobulin light chain.

When following a supervised machine learning approach it is assumed that there is a statistical relationship between certain input (features) and output (categorical or quantitative) variables, and that this relationship can be learned by models that are trained on representative data, and that these models can in turn predict the values of the output variable of interest from new input data. A subset of machine learning methods, collectively referred to as “deep learning,” is based on using model architectures consisting of several layers with units in them, that share some aspects of networks of neurons in the brain, thereby referred to as (deep) neural networks. The advantage of neural networks is that their ability to learn and predict is less dependent on the feature engineering process as compared to other types of machine learning algorithms, instead, neural networks can learn the important features by virtue of several data transformation steps. However, this typically requires large amounts of data.

Here, we specifically designed enrichment methods resulting in a large number of antibody sequences obtained by NGS, making it feasible to use deep learning algorithms using neural networks. Machine learning approaches have recently been applied to predict certain antibody properties [reviewed in (Miho et al., 2018; Graves et al., 2020; Marks and Deane, 2020)]; however, predicting a highly complex property such as polyspecificity has rarely been approached computationally. As opposed to previous low-throughput experimental assays to determine features of polyspecific antibodies, our unprecedented approach allowed us to predict polyspecificity and to determine features contributing to polyspecificity in a data-driven manner, which provides useful information to improve strategies for therapeutic antibody design in the future.

## 2 Methods

### 2.1 Experimental methods

#### 2.1.1 Transgenic rabbits

For immunization, Roche proprietary transgenic rabbits expressing a humanized antibody repertoire were used (Ros et al., 2020). Transgenic rabbits comprising a common human light chain are reported in WO2017/072208A1.

#### 2.1.2 Immunization

For each antigen, three rabbits were immunized with either human serum albumin (HSA), recombinant Fc-fused human TNFα or recombinant Fc-fused human TWEAK. Before the start of immunization blood was drawn for control (preimmune) blood samples. Animals were pre-stimulated with adjuvant and blood was taken after 7 days for adjuvant samples. For the first immunization with antigen, 400 µg protein was mixed with adjuvant and administered intradermally. One and 2 weeks later booster immunizations with 200 µg antigen mixed with adjuvant were given first intramuscular and then subcutaneously. Over a course of 3 months additional booster immunizations (each with 200 µg antigen) were given in a similar fashion every 4 weeks. For each immunogen and immunization step all animals were immunized at the same time of the day. Blood for immunized samples was taken ∼7 days post antigen immunizations, starting from the third antigen immunization onwards. For each immunogen and immunization step all blood samples were collected at the same time of the day, except for the 5th immunization, in which case blood samples were distributed over 3 days (1 animal per day) to allow for collection, B cell isolation, sorting and screening in a timely manner. Blood samples were used as a source of antigen-specific B cells in the B cell enrichment and the B cell cloning processes. The applied protocol was established during platform development in which titers were monitored regularly during the immunization period (see also, Seeber et al., 2014).

#### 2.1.3 Cell isolation and enrichment (panning)

For the isolation of peripheral blood mononuclear cells (PBMCs), blood samples containing EDTA were diluted two-fold with PBS, and were density centrifuged on Lympholyte Mammal (Cedarlane, CL5120) in Leucosep tubes (Greiner Bio-One, 227290). For enrichment of antigen-binding B cell clones (panning), a solution of the given antigen or polyantigen mix was prepared in carbonate buffer (0.1 M sodium bicarbonate, 34 mM disodiumhydrogencarbonate, pH 9.55) at a concentration of 2 μg/ml, and 2 ml/well of this solution was incubated on 6-well plates overnight at 4°C. Before enrichment, the coated plates were washed three times with PBS. In case of the antigens this solution contained only the given antigen, and in case of the polyantigens this solution contained the mix of the polyantigens (each at 2 μg/ml). The antigens used for the HSA, TNFα and TWEAK immunization groups were HSA (SIGMA, A9731), TNFα (Peprotech, 300-01A) and TWEAK (Peprotech, 310-06), respectively. The mix of polyantigens for the TNFα and the TWEAK immunization groups contained the following: Cardiolipin (SIGMA, C1649), dsDNA (Roche Diagnostics GmbH, Mannheim, Germany, 11467140001), KLH (SIGMA, H7017), Insulin (SIGMA, I0516), Lysozyme (SIGMA, L6876), LPS (SIGMA, L4516), Flagellin (InvivoGen, tlrl-epstfla-5), HSA (SIGMA, A9731). The mix of polyantigens for the HSA immunization group contained the same polyantigens except HSA. In order to eliminate monocytes binding to plastic (thereby occupying enrichment surface) as well as B cells that potentially bind plastic, PBMCs were incubated prior to enrichment in a cell culture flask for 1 h at 37°C (5% CO_2_) in cell culture medium (medium composition is described in (Seeber et al., 2014)). Then, non-binding cells were recovered, centrifuged and resuspended in cell culture medium and plated on the coated 6-well plates at 1.5 ml/well. For enrichment of (poly)antigen-binding B cells, these plates were incubated for 1 h at 37°C (5% CO_2_). To remove cells not binding the (poly)antigen, the supernatant was removed and the wells were washed with PBS. To collect the enriched cells binding the (poly)antigen, the plates were incubated with 0.5 ml trypsin solution per well for 5 min at 37°C (5% CO_2_), and washed twice with 1 ml medium.

#### 2.1.4 Next-generation sequencing

##### 2.1.4.1 RNA isolation

Total RNA from frozen cells was isolated using High Pure RNA Isolation Kit (Roche Diagnostics GmbH, Mannheim, Germany) according to the manufacturer’s instructions. RNA was eluted in 50 µL Elution Buffer, stored at −80°C, and handled on ice for further steps.

##### 2.1.4.2 cDNA synthesis

cDNA was produced by reverse transcriptase using an anchored-oligo(dT)_18_ primer from the Transcriptor High Fidelity cDNA Synthesis Kit (Roche Diagnostics GmbH, Mannheim, Germany). 20 µL RNA was used for a reverse transcriptase-reaction, for a final volume of 40 μL. 20 µL RNA, 2 µL oligo(dT) and 0.8 µL water were incubated for 10 min at 65°C and cooled down on ice. 8 µL Reaction Buffer, 1 µL RNase Inhibitor, 4 µL dNTPs, 2 µL DTT and 2.2 µL Transcriptase were added to the reaction solution and incubated at 50°C for 30 min, then 85°C for 5 min and finally cooled down on ice. Of each RNA sample two cDNA samples were prepared in parallel. The relative amount of specific rabbit IgG cDNA per sample was measured by ddPCR (BioRad) analysis. cDNA was stored at -20 °C until further processing.

##### 2.1.4.3 PCR amplification

From each cDNA sample, the variable regions of the heavy chain (V_H_) were amplified. A rabbit Fc PCR was generated: the forward primer was binding at the beginning of the leader region of VH (ATG​GAG​ACT​GGG​CTG​CGC​TGG​CTT​C) while the reverse primer at the beginning of the constant region of the rabbit heavy chain C_H_1 (GGG​AAG​ACT​GAT​GGA​GCC​TTA​GGT​TGC​C). PCR was done using AccuPrime Pfx SuperMix (Thermo Fisher) according to manufacturer’s instructions on a Veriti 96 Well Thermal Cycler (Applied Biosystems). The amount of cDNA template used in each PCR reaction was adjusted according to ddPCR results, in order to provide each reaction mix with the same amount of specific IgG cDNA copies.

PCR conditions for NGS are shown in Table 1.

**TABLE 1:** PCR conditions for NGS.

T°	Time	Cycles
94°C	4 min	1x
94°C	20 s	27x
68°C	20 s	
68°C	45 s	
68°C	4 min	1x
4°C	∞	1x

For each PBMC sample, two technical PCR replicates were made (derived from two cDNA replicates). Each PCR NGS template was generated from a pool of 8 (50 µL each) PCR reactions in order to achieve enough amount of template without raising PCR cycles. After amplification the aliquots were pooled and purified using the NuceloSpin Gel and PCR Clean-up kit (Macherey-Nagel), following the manufacturer’s instructions. DNA was eluted with 30 µL NE buffer.

##### 2.1.4.4 Library preparation

The DNA amplicons need specific indexes for Next-Generation Sequencing on the Illumina platform. Therefore sequencing-ready libraries were generated using the TruSeq Nano DNA Library Prep Kit for NeoPrep (Illumina) on the NeoPrep (Illumina) featuring the precision of digital microfluidics. All steps were done following manufacturer’s instructions without the step for DNA fragmentation. The SC550 for 550 bp inserts was used, since the amplicons have approximately a length of 450 bp. Normally 60 ng DNA in maximum of 15 µL (or filled up with H_2_O) was used and then mixed with the SC550 and DMB solution and further processed using six PCR cycles as recommended by the manufacturer. For samples with lower concentration, 15 µL of the sample were used while increasing the number of PCR cycles (up to nine cycles). Validation and normalization was not performed by the NeoPrep system. Libraries were then diluted to a concentration of 4 nM. DNA concentrations were measured by Qubit Fluorometer (Invitrogen) using the dsDNA BR Assay Kit or dsDNA HS Assay Kit.

MiSeq sample loading: Reagent cartridge was thawed according to the manufacturer’s protocol. 4 nM libraries were pooled (16 per sequencing run), denatured and diluted according to the manufacturer’s protocol to a final concentration of 13 pM. PhiX control was equally processed. At the end, 875 µL denatured 13 pM. library pool and 125 µL denatured 13 pM. PhiX were mixed, resulting in 12.5% PhiX in total. 600 µL were loaded into the appropriate reservoir of the cartridge. The run was started according to the MiSeq System Guide. MiSeq Reagent Kit v3 (600 cycles) was used for paired-end sequencing on the MiSeq (Illumina).

#### 2.1.5 Fluorescence-activated cell sorting (FACS)

For sorting of single B cell clones that express transgenic IgG, cells after antigen-enrichment (panning) were stained with anti-rabbit IgG-Fc-FITC (goat, AdB serotec, polyclonal) and anti-human IgΚ-APC (mouse, BD Pharmigen, monoclonal) in PBS [containing normal goat serum (Vector labs) and normal mouse serum (Southern Biotech) for blocking] for 30 min at 4°C in dark. Cells were then centrifuged and washed twice with ice-cold PBS. Cells were stained with 5 μg/ml propidium-iodide (BD Pharmigen) to identify live cells. Human IgG positive live B cells were sorted using a Becton Dickinson FACSAria with a FACSDiva software (BD Biosciences).

#### 2.1.6 ELISA

Rabbit IgG titer quantification of primary B cell supernatants was performed according to previously published standard methods (Seeber et al., 2014; Schrade et al., 2019). In brief, a mix of biotinylated mouse anti-rabbit IgG antibody and anti-rabbit IgG HRP conjugate was incubated for 90 min at room temperature on 384-well streptavidin-coated microtiter plates together with primary B cell supernatants diluted in PBS containing 0.5% BSA and 0.05% Tween-20. Next, the plates were washed repeatedly with PBS containing 0.2% Tween-20, HRP substrate solution was added to the plates and absorbance at 370 nm was measured.

Antigen and polyantigen binding ELISA was performed similarly to a previous publication (Schrade et al., 2019). In brief, for the binding assay the same antigens and mix of polyantigens were used as for cell enrichment (panning). 384-well plates were coated using these (poly)antigens at 2 μg/ml in PBS at 4°C overnight. Plates were washed repeatedly with PBS containing 0.1% Tween-20 between steps. Plates were blocked by incubation with PBS containing 2% BSA and 0.2% Tween-20 for 1 h at room temperature. Samples, i.e. primary B cell supernatants (primary screening) or solutions of purified recombinantly expressed antibodies with normalized IgG concentration (1 μg/ml, secondary screening) were diluted in PBS containing 0.5% BSA and 0.05% Tween-20, and were added to the coated and blocked plates, followed by a 1 h incubation at room temperature. A secondary anti-rabbit-IgG antibody (HRP conjugated) was added and incubated for 1 h at room temperature. HRP substrate solution was added and absorbance was measured at 370 nm to determine samples containing binding antibodies.

#### 2.1.7 PCR amplification of cognate VH and VK and recombinant expression

Total RNA preparation from B cells lysate (resuspended in RLT buffer, Qiagen, 79216) and cDNA synthesis were performed as described previously by Seeber et al. (Seeber et al., 2014).

The cDNA of the immunoglobulin heavy and light chain variable regions (heavy chain here called VH, kappa light chain here called VK) was amplified with the AccuPrime Supermix (Invitrogen, 12344-040) using the primer pairs rbHC.up and rbHC.do for the heavy chain and BcPCR_FHLC_leader.fw and BcPCR_huCkappa.rev for the light chain. All forward primers were specific for the signal peptide (of respectively VH and VK), whereas the reverse primers were specific for the constant regions (of respectively VH and VK). The PCR conditions for the heavy chains were as follows: Hot start at 94°C for 5 min; 35 cycles of 20 s at 94°C, 20 s at 70°C, 45 s at 68°C, and a final extension at 68°C for 7 min. The PCR conditions for the VKs were as follows: Hot start at 94°C for 5 min; 40 cycles of 20 s at 94°C, 20 s at 52°C, 45 s at 68°C, and a final extension at 68°C for 7 min. PCR products were cleaned using the NucleoSpin Extract II kit (Macherey-Nagel, 740609250) according to manufacturer’s protocol.

Primer sequences for the PCR amplification of cognate VH and VK are shown in Table 2.

**TABLE 2:** Primer sequences for the PCR amplification of cognate VH and VK.

Primer	Sequence
rbHC.up	AAG​CTT​GCC​ACC​ATG​GAG​ACT​GGG​CTG​CGC​TGG​CTT​C
rbHC.do	CCA​TTG​GTG​AGG​GTG​CCC​GAG
BcPCR_FHLC_leader.fw	ATG​GAC​ATG​AGG​GTC​CCC​GC
BcPCR_huCkappa.rev	GAT​TTC​AAC​TGC​TCA​TCA​GAT​GGC

For recombinant expression of monoclonal antibodies, PCR-products coding for VH or VK were cloned as cDNA into expression vectors by the overhang cloning method (Haun et al., 1992; Li and Elledge, 2007) and transiently co-transfected into HEK293 cells as described in Seeber et al. (Seeber et al., 2014). Two variants of the basic expression plasmid were used, one plasmid contained the rabbit IgG constant region and a second plasmid contained a human kappa-1 LC constant region designed to accept respectively the VH and VK regions. After purification by Protein A column standard protocols supernatants were further analyzed for antibody content and specificity.

### 2.2 Computational methods

#### 2.2.1 Data preprocessing

Paired-end sequencing reads were generated by Illumina MiSeq in fastq format. Quality control of sequencing reads was performed using FastQC (Andrews, 2010). Overlapping paired-end reads were merged using FLASH (Magoč and Salzberg, 2011) leading to one sequence (from each paired-end read) containing the antibody variable domain. An in-house developed algorithm (Klostermann, 2015) was used to extract the part of sequences that consist of exactly the variable domain as defined by the WolfGuy numbering scheme (Bujotzek et al., 2015). Subsequently, an output table was generated (as CSV file) containing the whole variable domain sequence and the individual antibody CDRs and framework regions, as well as the most similar germline-encoded gene segment (information not used as a feature for the model), identified by alignment to the IMGT germline reference sequences (Lefranc et al., 2005).

#### 2.2.2 Data partitioning for cross-validation and subsampling

For estimation of predictive performance and hyperparameter tuning, four types of data partitioning were used. For 10-fold cross-validation with random split, data was partitioned to 10 sets (folds) that were chosen randomly, of which, one was used as validation set and the rest as training set in a given round of cross-validation. For 10-times repeated subsampling, data was split into 10% validation set and 90% training set chosen randomly, and this was repeated 10 times independently (resulting in training sets with probable overlaps across the 10 rounds of subsampling). For blocked cross-validation using antigens or animals as grouping variable, data was partitioned to three or nine sets (folds) based on the immunization antigens or the animals, respectively, of which one set was used as validation set and the rest as training set in a given round of cross-validation. For all data partitioning methods, all possible overlaps between training and validation set (for example if a given antibody clone can be found in the immune repertoire of multiple animals) were explicitly eliminated, resulting in disjoint training and validation sets. Both for training and for validation, duplicates were also explicitly eliminated, i.e. unique sequence-sets were used.

#### 2.2.3 Feature encoding

For all analysis using the amino acid sequence of the VH region in this study, the VH sequences were represented in an aligned form using the WolfGuy numbering scheme (Bujotzek et al., 2015). Alignment gaps were represented by dots in the sequence. In total, two types of feature encoding was used in this study: (i) one-to-one mapping of letters in the VH sequence to integers was used for dimensionality reduction by UMAP only, and (ii) encoding of physico-chemical amino-acid features using a transformation by principal component analysis (PCA) was used for dimensionality reduction by UMAP as well as to generate input features for the classifier (i.e. feature encoding for the supervised learning part). For the encoding of physico-chemical amino-acid features, the feature set named “aaindex1” describing physico-chemical properties of amino acids was downloaded from https://www.genome.jp/aaindex/(Kawashima et al., 2007). By parsing this feature set, matrix M was generated with rows corresponding to amino acids and columns to physico-chemical features. PCA was performed on the column-scaled and column-centered M matrix by singular value decomposition. The first six principal components were used for feature encoding (of the total of twenty), that explain 79% of the variance. The principal component values corresponding to all possible amino acids were scaled and centered for each of the first six principal components. Each aligned VH sequence was encoded to a numeric feature vector by replacing each amino acid by its value on principal components 1-6 (scaled and centered), resulting in feature vectors of length 882 [147 (aligned sequence length) × 6 (number of principal components)]. Alignment gaps (dots) were encoded by replacing them with zero, and in case there were X-s (i.e. undetermined amino acids) these were encoded by median imputation, i.e. replacing them with the median value of all amino acids on the given principal component (scaled and centered). The feature corresponding to position i in the original VH sequence and principal component j from the transformation of physico-chemical descriptors is represented at element (((j-1) × 147)+*i*) of the resulting feature vector. This encoding resulted in a representation of the amino acid sequence that retains positional information in the VH region and contains relevant physico-chemical information in a compressed form.

#### 2.2.4 Dimensionality reduction by UMAP

The feature-encoded VH sequences were used as input to the Uniform Manifold Approximation and Projection for Dimension Reduction (UMAP) (McInnes et al., 2018) algorithm (unsupervised dimensionality reduction). The UMAP implementation available in the uwot package was used (implemented in R and C++). An appropriate distance metric was chosen for each feature-encoding method (Hamming distance for the one-to-one mapping from letters to integers; Euclidean distance for the encoding using PCA of physico-chemical properties). The following hyperparameter settings were used for UMAP when using the one-to-one mapping from letters to integers as feature-encoding method: n_neighbours = 1890 (3.5% of the number of observations), n_components = 2, metric = “hamming”, n_epochs = 500, spread = 4.75, min_dist = 0.65, fast_sgd = F. The following hyperparameter settings were used for UMAP when using the encoding using PCA of physico-chemical properties as feature-encoding method: n_neighbours = 3780 (7% of the number of observations), n_components = 2, metric = “euclidean”, n_epochs = 500, spread = 5.75, min_dist = 0.15, fast_sgd = F. A random subset of the entire data set was used for UMAP by randomly sampling 500 clones per experimental condition, where a given experimental condition corresponds to a certain animal, antigen, time-point and enrichment.

#### 2.2.5 Model architecture

For building neural networks, the Keras library was used with the TensorFlow backend. For the input layer of the model, the feature-encoded VH sequences were used (see previous section). The final model is a neural network containing three fully connected hidden layers (with 6, 3, and 2 units, respectively) each followed by a batch normalization layer, an Exponential Linear Unit (ELU) activation layer (alpha = 1), and a dropout layer (dropout rate = 0.5). For the output, a fully connected layer with a single unit and sigmoid activation function was used.

#### 2.2.6 Training

Binary cross-entropy loss was used as an objective function to be minimized during training. As an optimizer, the Adam optimizer was used (Kingma and Ba, 2017). For the final model a learning rate of 6 × 10^−7^ was used, with a batch size of 128 applying a balanced minibatch sampling such that in each round of the back-propagation the same number of observations were drawn randomly from both classes (64 antigen-specific, 64 polyspecific clones), and the training was performed for 10 epochs.

#### 2.2.7 Performance evaluation

Several performance metrics were considered collectively for the evaluation of cross-validation and subsampling, namely: binary cross-entropy loss, area under ROC curve, F1, MCC, sensitivity and specificity. The following hyperparameters were optimized: model architecture i.e. number of layers and number of units in each layer, learning rate, batch size, dropout rate. Results from the blocked cross-validation using antigen-based data partitioning were considered as the main performance indicator.

#### 2.2.8 Variable importance calculation

Variable importance was calculated using the method described by Fisher and others (Fisher et al., 2019). The variable importance was estimated using a subsampling and permutation approach. In one round of the subsampling procedure, 3000 clones were resampled randomly such that the two classes are balanced (i.e. sampling 1,500 antigen-specific and 1,500 polyspecific clones). The VH sequences of these clones were encoded to the features used by the model, and the probability of polyspecificity was predicted by the model using the feature values corresponding to these observations (clones) in an unpermuted and a permuted fashion (permutation of values across observations was performed for each feature separately). Using the probability values predicted by the model and the true labels available from the data, the binary cross-entropy loss was calculated for the unpermuted case and the permuted cases. The ratio of the permuted and unpermuted loss values was considered as a measure of variable importance (dropout loss ratio). In each round of resampling, the permutation of feature values and the calculation of dropout loss ratio was repeated three times, and the mean of the calculated dropout loss ratio was considered as the result of a given resampling. The resampling procedure was repeated 1,000 times resulting in 1,000 dropout loss ratio values per feature. For visualization in the figures, the mean of these values was used. To estimate the statistical significance of the importance of each feature, one-tailed *p*-values were calculated using the dropout loss ratio as test statistic using a non-parametric approach. The null hypothesis was defined as the case that the loss calculated with permutation of the feature values is less than or equal to the loss calculated without permutation of the feature values, and therefore the dropout loss ratio is less than or equal to one (implying that the given feature is not important for the model’s prediction). The *p*-value was calculated as the fraction of resampling rounds where the dropout loss ratio was less than or equal to one. Following the calculation of *p*-values for each feature, False Discovery Rates (FDR) were calculated from the *p*-values using the Benjamini and Hochberg procedure (Benjamini and Hochberg, 1995) to correct for multiple testing across the features. In addition to calculating importance of features used by the model, a back-calculation of importance was also performed to quantify the importance of the physico-chemical features that were used for the PCA-based feature encoding. The back-calculation was carried out separately for each initial physico-chemical feature (prior to transformation by PCA) and for each position at the VH sequence. For this, the dropout loss ratio of each feature used by the classifier (each corresponding to a given position and principal component) and the loadings (weights in the linear combination) of each initial physico-chemical input feature on the first six principal components were used. The following equation was used for the back calculation with i and j referring to the principal components:

$$back–calculated\;importance=\sum_{i=1}^{6}{dropout\;loss\;ratio}_{i}\times \frac{{loading}_{i}}{\sum_{j=1}^{6}{loading}_{j}}$$

Subsequently, applying the same calculation procedure as for the features directly used by the model, the means of the back-calculated importance values were used for visualization, and FDR was considered as a measure of statistical significance.

## 3 Results

Despite the detrimental impact of polyspecificity in the development of therapeutic antibody-based drugs, currently there is no method available that can predict polyspecificity from the sequence of the antibody. Accordingly, the antibody features influencing polyspecificity are poorly understood. Prior structural calculations can predict binding to some extent but are computationally expensive and not applicable to large-scale. To predict polyspecificity and to gain insight into the key features affecting it, herein we pursued a large-scale data-driven approach using next-generation sequencing data of antibody VH domains in an immunization model including specificity-based enrichment of binders combined with deep learning.

### 3.1 Identification of polyspecific antibody clones using binding enrichment

We considered polyspecificity prediction as a binary classification problem, statistically modeling the relationship between the specificity and the sequence of antibodies. In this setup, the binary target variable to be predicted is the specificity of an antibody, with “polyspecific” and “antigen-specific” being the two classes, and the predictors are features derived from the amino acid sequence of the heavy chain variable region. In order to train machine-learning-based models using this setup, antibody sequence data (features) and the corresponding binding specificity (labels) are needed. Therefore, we generated a suitable data set using immunization of transgenic rabbits with different model antigens, followed by enrichment of immune cells based on their binding specificity in order to assign specificity labels to the correspondingly derived sequences.

**FIGURE 1:**
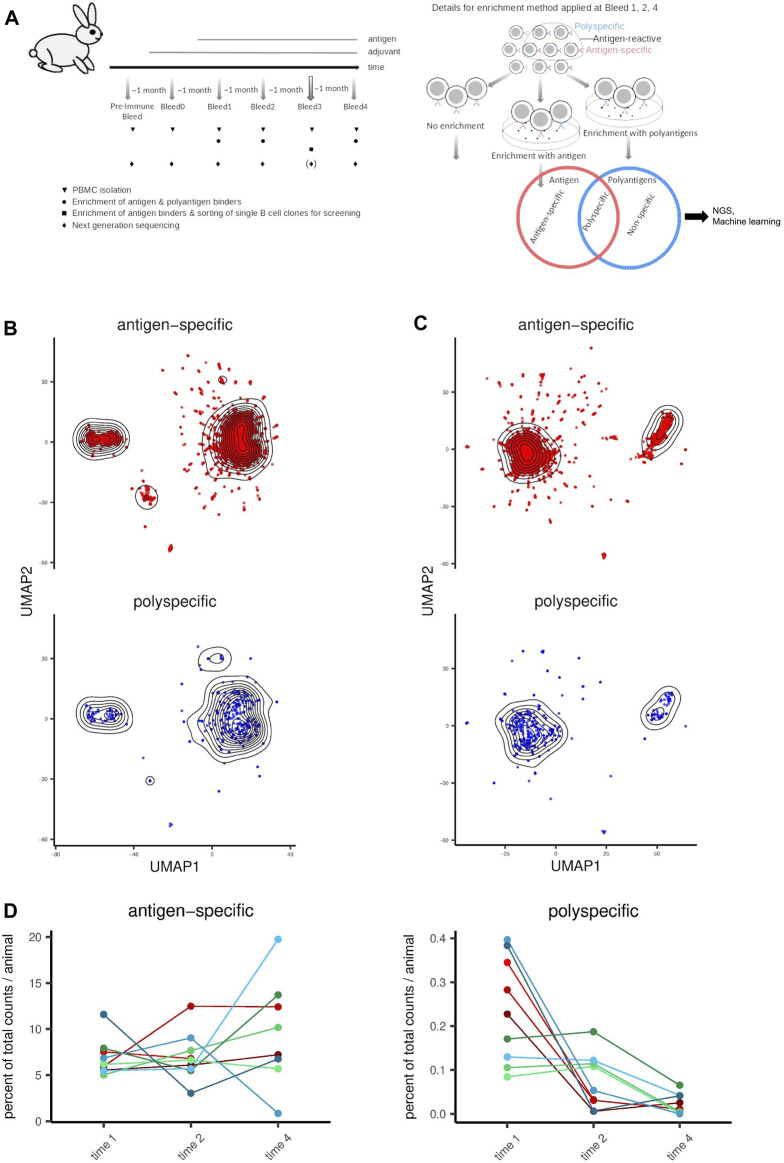
Distribution and similarity of polyspecific and antigen-specific antibody clones in relation to experimental design parameters. **(A)** Schematic representation of the experimental design. The left panel shows the timelines for immunization, sampling and enrichment. On the right panel, details for enrichment are shown. Red denotes antigen which is either HSA, TNFα or TWEAK depending on the group of animals. Blue represents polyantigen mixture, which was a mixture of Cardiolipin, dsDNA, KLH, Insulin, Lysozyme, LPS, Flagellin, and (except for the HSA group) HSA. For each of the three antigen groups, blood samples from three animals per group were taken at each timepoint. **(B)** Similarity of antibody clone sequences is visualized using UMAP for dimensionality reduction. Sequences encoded to integers were used as input with Hamming distance as similarity metric. **(C)** Visualization as in **(B)**. Sequences encoded to PCA-based physico-chemical feature vectors were used as input with Euclidean distance as similarity metric. **(D)** Distribution of polyspecific and antigen-specific clones across time-points and animals shown as percent of all unique sequences per animal. Individual lines represent separate animals. Immunization groups are represented as follows. Red: HSA, green: TNFα, blue: TWEAK.

To generate antigen-specific and polyspecific antibodies, we used three different model antigens for immunization: human serum albumin (HSA), tumor necrosis factor alpha (TNFα), and TNF-related weak inducer of apoptosis (TWEAK) with one antigen per animal and three animals per antigen. We collected samples for RNA-seq of the antibody heavy chain variable region (immunoglobulin VH region) at four time points after immunization with the antigen (bleed 1-4; ∼1 month between each time point; Figure 1A and Supplementary Figure S1A). This design largely covers the time course of the adaptive immune response from early until late (affinity matured) antibodies in our sampling. In this way, we identified ∼52 million unique VH sequences in total. Per sequencing sample, 256882 (mean; SD = 131395) unique protein sequences were obtained, and lysates from each experimental condition were split into two sequencing samples (technical duplicates) from which the resulting data was pooled before further analysis. We also collected samples from the pre-immune and bleed 0 time points, respectively, to identify clones present in the naïve immune repertoire (pre-immune, time point before any immunization) or clones that arise as a consequence of applying the immunization adjuvant alone (bleed 0). Clones observed at these time points, as well as clones that were shared between any of the three immunization groups (antigens) regardless of time point, are likely independent of the immunization and therefore were excluded from the supervised machine learning model training and cross-validation. Instead, this set of clones was considered separately as a test (holdout) set.

In order to identify B cell clones producing antigen-specific or polyspecific antibodies (i.e. expressing such B cell receptors), we enriched the binding B cell population (at bleed 1, 2, and 4) using the panning technique (Wysocki and Sato, 1978) separately for (i) the antigen that was used for the immunization and (ii) a set of polyantigens that we carefully selected for the panning procedure with a high likelihood to reflect immunoglobulin and not cellular unspecific binding, and have been described in the literature to be frequently bound by polyspecific antibodies (Wardemann et al., 2003; Mouquet et al., 2010; Andrews et al., 2015; Bunker et al., 2017; Prigent et al., 2018; Planchais et al., 2019). We defined antibodies to be antigen-specific if they bind only the antigen, and polyspecific if they bind both the antigen and, in addition, structurally unrelated molecules (polyantigens; Supplementary Figure S1A). Therefore, we assigned specificity labels to the antibody clones such that clones enriched by the antigen only belong to the antigen-specific class and those enriched by both the antigen and the set of polyantigens belong to the polyspecific class. In parallel, we collected data from the non-enriched cell population as a representation of the B cell repertoire of the given animal at the respective time point. To also assign time labels to the antibody clones, each clone was assigned the time point at which it was first observed.

To generate a large amount of data from millions of antibody clones in parallel, bulk (cell population) sequencing is more suitable than single cell sequencing. However, with a bulk sequencing approach, it is not possible to identify the pairs of heavy and light chain (VH and VL sequences) that belong to the same antibody clone. Therefore, we focused our analysis on the VH region, which plays a key role in antibody-antigen binding and provides the largest diversity owing to its genetic make up. In order to eliminate the variability in terms of binding that is caused by the different VL regions across antibody clones, and to keep the binding contribution of the VL region as a constant factor, we immunized rabbits transgenic for the human immunoglobulin locus expressing the same VL region (common-light chain) across antibody clones.

### 3.2 Polyspecific antibodies are rare and do not have a distinct set of VH regions

With our approach (Supplementary Figures S1A, B), we assigned ∼15 million and ∼0.134 million unique VH sequences to the antigen-specific and polyspecific classes, respectively. To get an unbiased view of the generated data set, we applied a dimensionality reduction approach for data exploration. We sampled the same number of clones (500 clones) randomly from each experimental condition and applied Uniform Manifold Approximation and Projection (UMAP) (McInnes et al., 2018) to project the clones to a two-dimensional space based on their sequence, visualizing the similarity between clones. We carried out this procedure applying two different feature-encoding methods prior to UMAP, and accordingly, two different distance metrics for UMAP: (i) for sequence similarity we encoded the aligned amino acid sequences using a one-to-one mapping from letters to integers and used the Hamming distance as distance metric (Figure 1B), and (ii) for similarity in terms of physico-chemical properties, we encoded the aligned amino acid sequences to PCA-based physico-chemical descriptors and used Euclidean distance as a distance metric (Figure 1C and Supplementary Figure S2).

Interestingly, at the overall repertoire level and when visualizing similarity properties accordingly, we observed an animal specific pattern which was preserved over time (Supplementary Figures S3A, B). This was also confirmed using Jaccard index of shared VH and CDR3 sequences as a similarity metric (Supplementary Figure S5). The adjuvant alone had no profound effect on the repertoire distribution as evident from the comparison of preimmune and time 0 time points (Supplementary Figures S3A, B).

The polyspecific clones did not form a separate cluster from the antigen-specific clones, indicating that they are not majorly distinct, at least when considering their VH region as a whole (Figures 1B, C, Supplementary Figures S3C, D). This corroborates the concept that prediction methods must capture a large complexity of antibody features, as polyspecific antibodies did not share easy-to-detect paramount features that other antibodies lack. Furthermore, in each animal, the number of polyspecific clones was much lower than the number of antigen-specific clones (with an average polyspecific:antigen-specific antibody ratio of 1:113), highlighting also the efficiency of antigen immunization. Furthermore, most polyspecific clones were first observed at early time points in the immune response (bleed 1–2) in contrast to antigen-specific clones (bleed 4; Figure 1D, Supplementary Figures S3C, D), which supports the notion that antigen-specificity is increased throughout affinity maturation (Shehata et al., 2019).

### 3.3 Neural-network-based prediction of polyspecificity

In order to train a model predicting polyspecificity, the specificity label was used as target variable and the features resulting from encoding the VH sequence were used as predictors (see above and methods section). The polyspecific class was considered as positive class and the antigen-specific class as negative class. Because polyspecific antibodies were much rarer than specific antibodies, this class imbalance was corrected for during model training. We chose a neural-network-based approach to predict polyspecificity in order to be able to model complex non-linear relationships.

**FIGURE 2:**
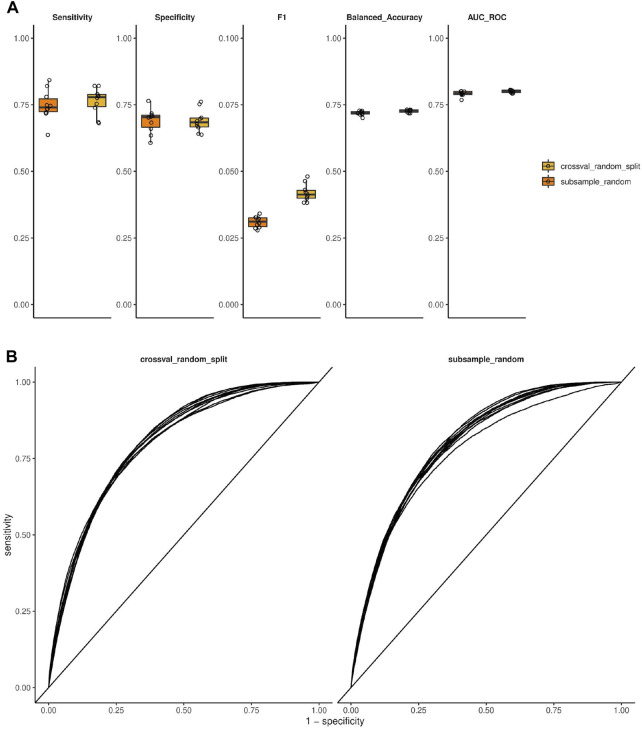
Performance estimation of models predicting antibody polyspecificity using cross-validation and subsampling. **(A)** Performance metrics are shown as a summary of a 10-fold cross-validation with random splitting or random subsampling repeated 10 times as indicated. **(B)** ROC curves resulting from the same setup and data as in **(A)**.

To estimate the prediction performance of our modeling setup, we applied cross-validation with various ways of data splitting as well as subsampling, and used several performance metrics. Using 10-fold cross-validation with random data splitting, the models were able to predict polyspecificity with an area under ROC curve of 0.800 (mean; SD = 0.005), sensitivity of 0.763 (mean; SD = 0.049), and specificity of 0.690 (mean; SD = 0.041) (applying a probability cutoff of 0.5). The 10 times repeated subsampling resulted in an area under ROC curve of 0.792 (mean; SD = 0.010), a sensitivity of 0.748 (mean; SD = 0.057), and specificity 0.689 (mean; SD = 0.045) (Figure 2).

To also estimate the prediction performance using a more stringent data splitting scenario, we also quantified these performance metrics using a blocked cross-validation approach, whereby data is split into folds based on a grouping variable. In this case the splitting variable was either the animal or the antigen (immunization group) that the clone originates from, resulting in a 9-fold or 3-fold cross-validation, respectively (9 animals, three antigens). Cross-validation with animal-based data splitting resulted in an area under ROC curve of 0.668 (mean; SD = 0.062), a sensitivity of 0.624 (mean; SD = 0.162), and specificity of 0.605 (mean; SD = 0.094). Cross-validation with antigen-based data splitting resulted in an area under ROC curve of 0.634 (mean; SD = 0.020), a sensitivity of 0.487 (mean; SD = 0.070), and specificity of 0.677 (mean; SD = 0.058) (Supplementary Figure S6).

**FIGURE 3:**
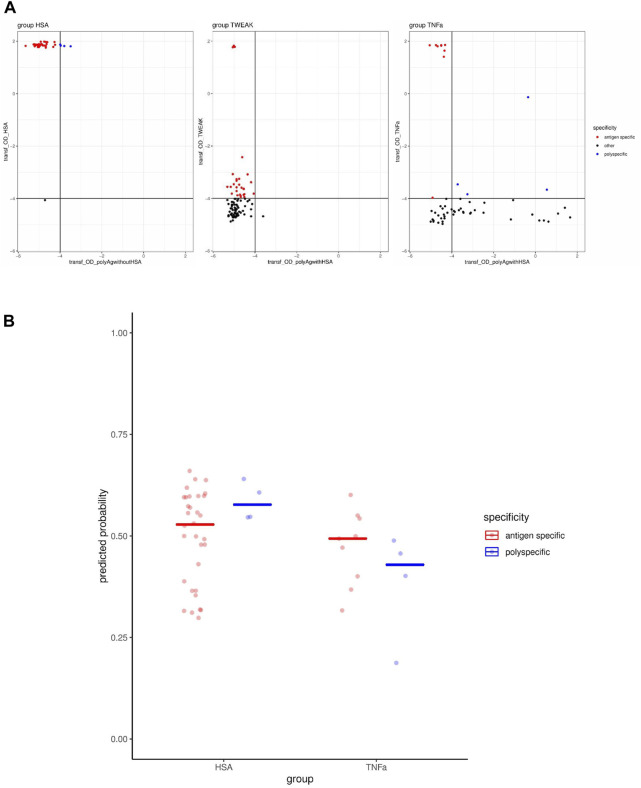
Performance testing of model predicting polyreactivity using screening data from bleed 3. **(A)** Transformed OD values, using a log2(OD + 0.05) transformation, are shown from ELISA for binding to the mix of polyantigens and the immunization antigen in each immunization group. The specificity labels assigned to each clone are indicated by the colors. **(B)** Probability of polyspecificity predicted by the model for antibody clones assigned to antigen-specific or polyspecific classes using screening data from bleed 3.

In order to estimate the model performance in a setting where polyspecificity is defined unambiguously for each individual clone by a separate measurement, instead of enrichments on the clone-population level, we generated a data set for this purpose. To this end, single cells were sorted from bleed 3 and the primary supernatants were screened by ELISA for IgG antibodies that bind the immunization antigen or the set of polyantigens. For a selected set of antibody clones, the IgG molecules were recombinantly expressed and subjected to a secondary screening using the same (normalized) IgG concentration to confirm binding to the antigen as well as to the set of polyantigens. In this way, we identified several antigen-specific clones that bind exclusively the antigen, as well as several clones that bind exclusively the polyantigen mix. However, we detected only a very low number of polyspecific clones (HSA group: four clones, TNFα group: four clones, TWEAK group: 0 clones) that bind both the antigen and the set of polyantigens (Figure 3A), which is in line with the observed low prevalence of polyspecific clones especially considering affinity matured clones (Figure 1D). Nevertheless, we tested whether our model would predict a higher probability of polyspecificity for the clones that we determined to be polyspecific compared to those identified as antigen-specific. In the HSA group, where the OD values were in a similar range with respect to the antigen-binding axes for the polyspecific and antigen-specific clones, the predicted probabilities of polyspecificity were higher for the clones labeled as polyspecific than for those labeled as antigen-specific, matching the expectation (Figures 3A, B). However, in the TNFα group, the predicted probability of polyspecificity was not higher for the polyspecific than for the antigen-specific clones (Figure 3B), which may be due to overall lower OD values on the antigen binding axes for the polyspecific clones (Figure 3A). Overall, this data set of selected clones might be too limited in size to assess the performance of our model. Applying a higher OD threshold on the antigen binding axes could result in a more precise assignment of polyspecificity labels, and at the same time, would leave only the HSA group containing any polyspecific clones. Although the performance estimates may be inaccurate due to the small sample size, calculated performance metrics were as follows: Sensitivity 1 and 0, Specificity 0.47 and 0.67, area under ROC curve 0.73 and 0.28, balanced accuracy 0.73 and 0.33 for the HSA and TNFα groups, respectively.

As an additional test set to estimate the model performance, we also used the NGS-derived VH sequences that were excluded from the training set (because they were observed before immunization with the antigen and/or because they were shared between any of the immunization groups). The “antigen-specific” or “polyspecific” labels were assigned to these sequences based on the enrichments in the same way as for the training set, and several performance metrics were quantified using these labels and the model’s predictions. All the different metrics showed a performance greater than randomly expected across most of the sequence sets (Supplementary Figure S7).

### 3.4 Identification of relevant features influencing polyspecificity

Next, we intended to determine which physico-chemical features are crucial contributors to polyspecificity of antibodies. To this end, we investigated the importance of each feature for our model using the permutation-based variable importance calculation approach (Fisher et al., 2019). This method is based on the assumption that, if a given feature is important, then permuting its values across observations will worsen the model’s ability to correctly predict, and therefore will lead to an increased loss function output. Therefore, using the NGS-based data set of polyspecific and antigen-specific VH sequences that was used for the training of the neural network, we calculated the ratio of the loss using the permuted and unpermuted values (dropout loss ratio) of each feature. The permutation was carried out for each feature on 1,000 sets of sequences obtained by subsampling. The mean dropout loss ratio was considered as a summarized measure of variable importance, and FDR was used to assess statistical significance. The features we used as input for this analysis were the same features as those used for the training of the model, i.e. they correspond to the combinations of amino acid positions in the VH sequence and principal components from the transformation of physico-chemical variables. Most of the features that were found to be significantly important for the model’s prediction were located in the Framework3 region, followed by the CDR3 region (Supplementary Figure S8).

**FIGURE 4:**
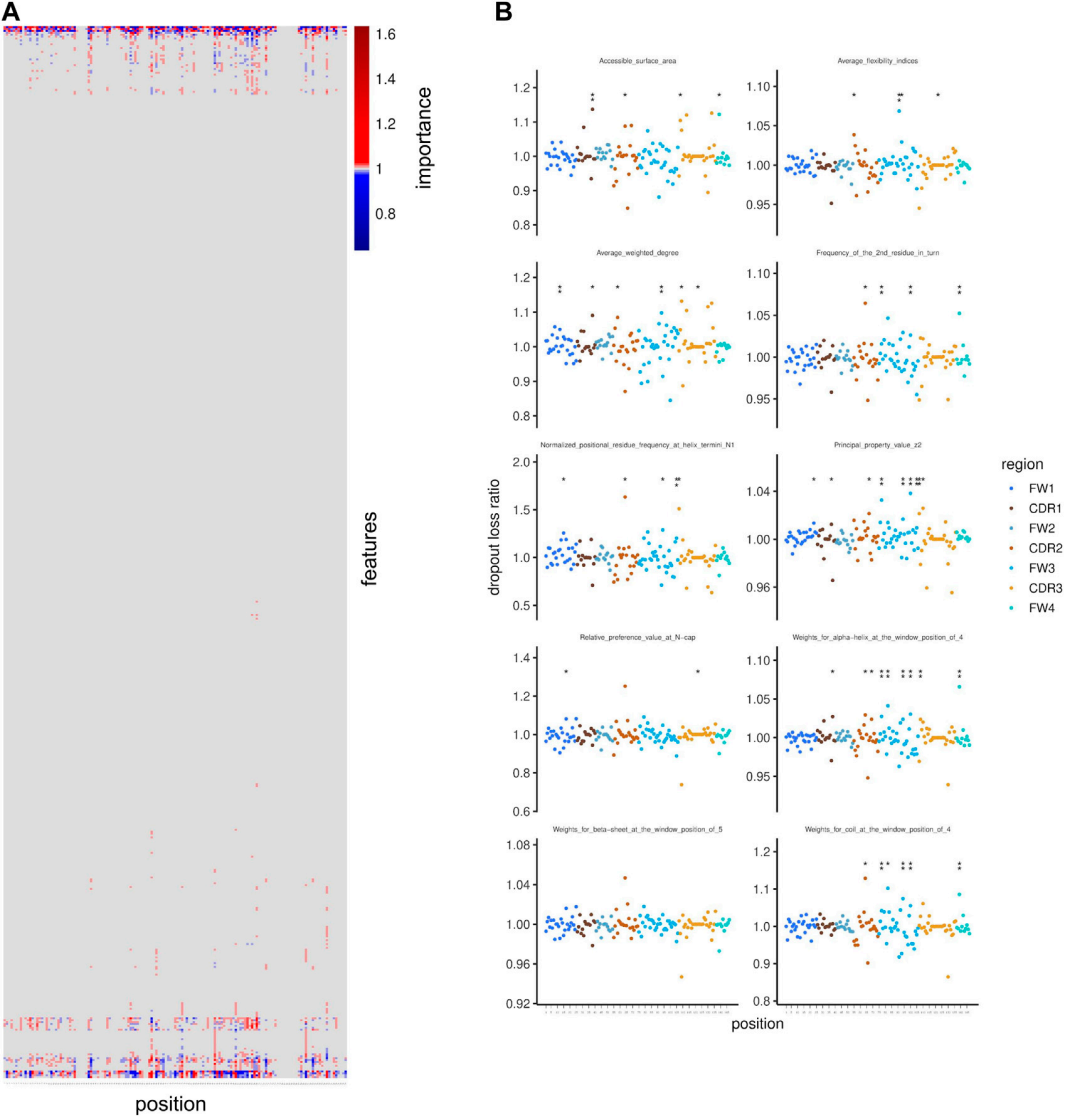
Feature importance for polyspecificity prediction. **(A)** Importance of amino acid physico-chemical features is shown along positions in the VH region. Importance for polyspecificity prediction is indicated by values above 1. **(B)** Importance is shown per position for amino acid physico-chemical features that were selected as the 10 highest ranked features when considering the maximum importance value across positions per feature for ranking in a descending order. Dots represent the mean values across resampling rounds and stars indicate statistical significance based on FDR (* indicates FDR < 0.1; ** indicates FDR < 0.05).

In order to interpret the importance of each position expressed in terms of the initial physico-chemical features prior to transformation by PCA, we carried out a back-calculation using the dropout loss ratios and the loadings on the principal components, resulting in importance values that correspond to the combinations of amino acid positions and the initial physico-chemical properties (for example charge or hydrophobicity at a given position). This analysis revealed that a few properties are more important than the rest and they can be grouped based on the positions at which they are important (Figure 4A). We focused on the ten most important physico-chemical properties that we defined as those ranked in the first ten when ranking the physico-chemical properties in descending order based on their maximum importance across all positions. For example, accessible surface area (Radzicka and Wolfenden, 1988), average flexibility indices (Bhaskaran and Ponnuswamy, 1988) and average weighted degree (Kakraba and Knisley, 2016) showed high importance, to which the CDR regions significantly contributed (Figure 4B).

**FIGURE 5:**
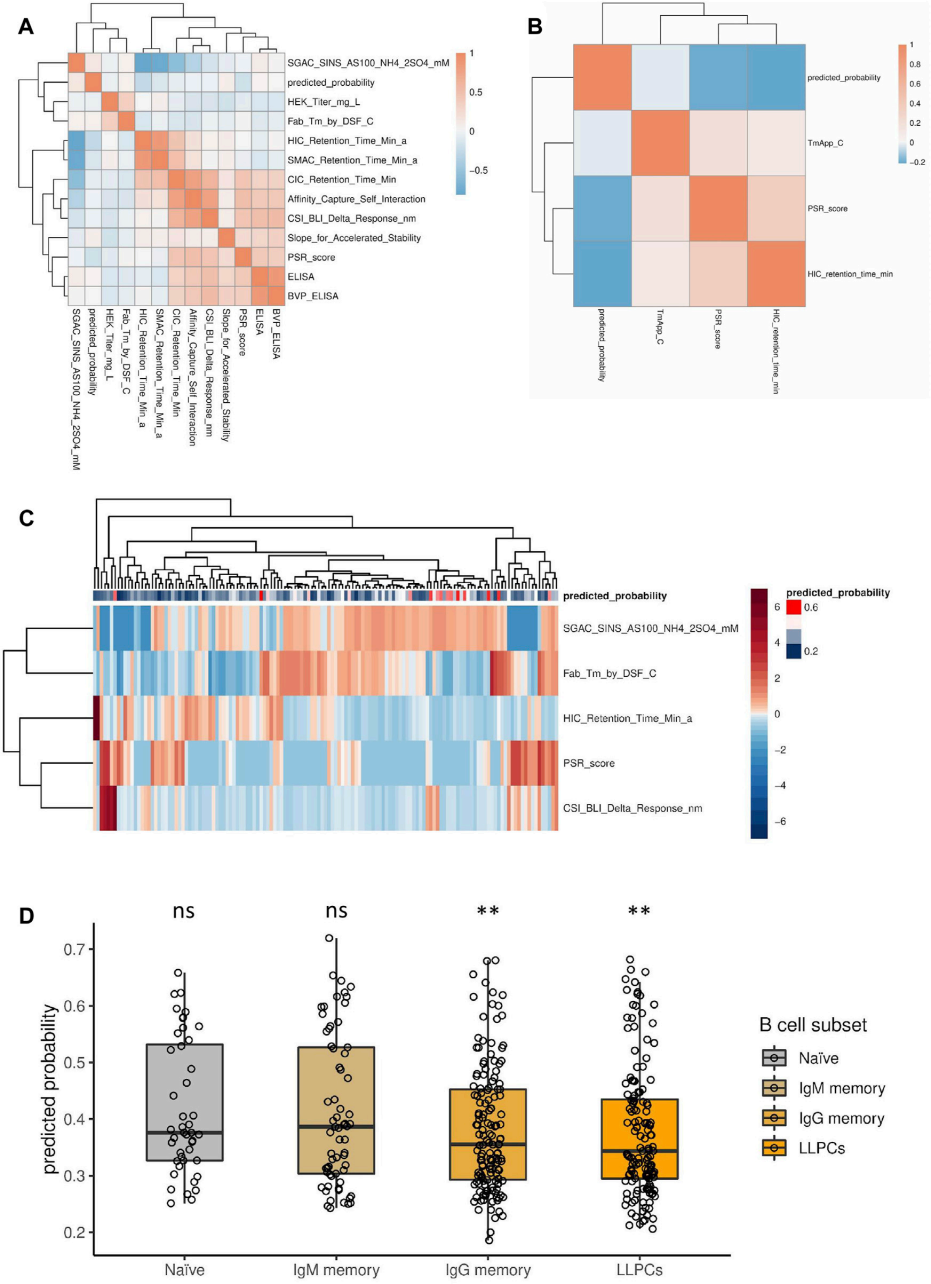
Association between physico-chemical properties of antibodies and predicted probability of polyspecificity using published data sets. **(A, B)** Spearman correlation between measured physico-chemical properties and the predicted probability of polyspecificity using the data set from Jain et al. **(A)** and Shehata et al. **(B)**. **(C)** A subset of physico-chemical properties (rows) were selected from **(A)**, based on evaluating distances in the feature space along with the predicted probability of polyspecificity, and were used for clustering the individual antibodies (columns). Antibodies are annotated based on their predicted probability of polyspecificity, as depicted by the color scale. **(D)** Probability of polyspecificity predicted by the model for antibodies at different maturation stages using the data set from Shehata et al. Significance was calculated by logistic regression. ns denotes not significant; ** denotes *p*-value < 0.005.

To test if there are any emergent physico-chemical properties of antibodies that correlate with the prediction of our model, we used independent published data sets. We considered two data sets, from the publications of Jain, Shehata and their colleagues (Jain et al., 2017; Shehata et al., 2019), since in these studies the sequence and numerous experimentally measured physico-chemical properties of the antibodies were provided, making them suitable for this analysis. First, the VH sequences from these data sets were encoded into the features that our model uses by converting them to an aligned representation (with the alignment gaps) and recoding the amino acid letters to the corresponding PCA transformed physico-chemical properties. These feature-encoded sequences were used as input to our model to predict the probability of polyspecificity of these clones. Then, the association between the predicted polyspecificity probability and the measured properties was quantified using Spearman’s rank correlation coefficient as a measure of association. To eliminate observations that contain no information, observation pairs with any missing values or zeros were excluded from the correlation calculation. Most measured physico-chemical properties only weakly correlated with the predicted probability of polyspecificity individually, with some actually displaying a negative correlation with the predicted probability of polyspecificity in both data sets (HIC retention time and PSR score; Figures 5A, B, Supplementary Figures S9, S10). While individual properties did not unequivocally coincide with predicted polyspecificity, it is plausible that a combination of measured physico-chemical properties could do so. Indeed, when clustering antibodies based on a subset of these properties, most antibodies that were predicted to be polyspecific clustered together, indicating that multiple physico-chemical properties in combination might have a role in polyspecificity (Figure 5C).

Furthermore, we investigated whether the predicted probability of polyspecificity would be different between antibodies originating from B cell clones at different stages of maturation, i.e. from naive to affinity matured clones, using the data set from Shehata and others (Shehata et al., 2019). Consistent with the notion that affinity maturation increases specificity, the predicted probability of polyspecificity was generally lower for affinity matured clones compared to naive or early clones (Figure 5D) (Shehata et al., 2019).

In summary, we characterized polyspecific antibodies using a novel strategy of combining the outcome of B cell enrichments by different (poly)antigens with sequencing data of these antibodies in the course of an immune response. Subsequently, we created a neural-network-based model that predicts the probability of polyspecificity from the VH sequence, and identified features that may influence the polyspecificity of antibodies.

## 4 Discussion

We developed a neural-network-based model using NGS data, specifically immunoglobulin VH sequences, that could predict polyspecificity of antibodies as confirmed by cross-validation. This unique and novel approach uses a large-scale data-driven method to predict polyspecificity using machine learning techniques. With our method, we achieved a sensitivity of around 75% and a specificity of around 70% along with an area under ROC curve of around 80%, which appears as a sound predictive power, given the complexity of the task as well as the limitation of the input to solely the VH sequence. Since there was a class imbalance in the data (few polyspecific and many antigen-specific antibodies), we used a balanced minibatch sampling in the training of the model to obtain a model that is not affected by the class imbalance. Of note, the above mentioned metrics are not sensitive to class imbalance. These values were obtained using a random subsampling or cross-validation with random split, as most commonly done in the machine learning field. Another commonly used metric that incorporates precision is the F1 score, which is sensitive to class imbalance. The F1 score calculated using our model was around 0.042 and 0.031, using cross-validation with random split and random subsampling, respectively. These F1 score values are better than the baselines of 0.017 and 0.013, respectively, that would be expected by a random classifier, with the given class imbalance and the given sampling. To challenge our modeling setup with a more realistic and stringent data splitting into training and validation data, we also applied splitting by animal or antigen (Supplementary Figure S11), which more realistically reflects the application of the model to a new and unrelated data set of interest. One assumption of training and validation in machine learning is that the training and validation data come from the same distribution. Even though this assumption does not apply when splitting the data for cross-validation based on animals or antigens, creating a conceptually difficult learning and prediction task, our method was still predictive for polyspecificity, albeit with lower performance as compared to the random split.

In addition, we compared the prediction of our model with available sequence data sets of antibodies related to polyspecificity readouts. One common feature we found associated to polyspecificity in these published data sets was hydrophobicity, and indeed this feature is also correlated with other undesirable properties of therapeutic antibodies, such as aggregation and precipitation (Tsai and Nussinov, 1997; Chennamsetty et al., 2009). Interestingly, in the case of broadly neutralizing antibodies against HIV, polyspecificity involves hydrophobic interactions and conformational plasticity (Prigent et al., 2018). It is noteworthy that hydrophobicity was associated with polyspecificity across different data sets despite the fact that different species, immunization antigens and assays (non-specific polyreactivity enrichment antigens) were used for determining polyreactivity, that are not directly comparable. Interestingly, in another recent study using camelid antibody fragment (nanobody) libraries with enrichment of polyreactive nanobodies on insect membranes and subsequent machine learning to predict features associated with polyreactivity, hydrophobicity was not strongly predictive of polyreactivity (Harvey et al., 2022). Thus, the type of antibody and the type or mixture of antigen(s) used may result in different features correlating with polyspecificity. For example, a certain antigen used for immunization or coating in binding assays might lead to a different outcome based on the properties of a certain specific antigen that may not be shared with all other antigens used to determine polyspecificity and hence may differ depending on the detailed antigen mixtures and cutoffs used in binding assays. Given these considerations about the influence of the specific antigen of interest, obtaining a single universally true prediction about the polyreactivity of all antibodies remains a challenge, and availability of more data sets of polyspecific antibodies may help improving models and identifying universally important features of polyspecificity in the future.

While certain physico-chemical properties may allow for predicting the probability of an antibody sequence to be polyspecific, much remains to be understood about the specific properties determining this characteristic. Adding to the complexity, it has been proposed that there might be several mechanisms, thereby several subtypes, of polyspecificity, such as the mechanisms of induced fit, conformational isomerism, rigid adaptation, or differential ligand or epitope positioning [reviewed in (Dimitrov et al., 2013; Regenmortel, 2014)]. In our enrichment experiments, we used a set of polyantigens in order to capture as many mechanisms of polyspecificity as possible using one assay. Despite containing several molecules for enrichment, this assay might only capture certain mechanism(s) of polyspecificity, but most likely not all possible mechanisms. The difference in terms of assay components that are used to determine polyspecificity might provide an explanation for the discordance between different readouts (such as the weak inverse correlation between our model’s prediction of polyspecificity and the PSR assay from (Jain et al., 2017; Shehata et al., 2019)).

Regarding specific amino acids overrepresented in polyspecific antibodies, different conclusions have been made in the literature. For example, polyreactive antibodies have been suggested to have long H3 regions that are rich in tyrosine and tryptophan (Aguilera et al., 2001). However, using synthetic phage display libraries, tyrosine content has been shown to correlate with specificity, while content of arginine (that is positively charged at physiological pH) correlated with polyspecificity (Birtalan et al., 2008). The length of CDRs was shown to correlate with hydrophobicity and aggregation, and positively or negatively charged residues in the CDR H2 region were beneficial for developability of antibodies. In detail, (i) tryptophan-rich CDR H3 loops were associated with binding promiscuity (measured by PSR assay), hydrophobicity and self-aggregation, (ii) a high frequency of aliphatic amino acids in the CDR H3 region was suggested to contribute to polyreactivity, and (iii) glycine frequency in the CDR H3 region was associated with hydrophobicity (Lecerf et al., 2019). In another study, epitopes of polyreactive antibodies have been described as proline-rich, and since proline is often present in loops and turns (solvent-exposed sites), it might contribute to a conformation-influencing factor in epitopes of polyreactive antibodies (Tchernychev et al., 1997). It has been proposed that polyspecificity is driven by conformational flexibility and less specific intermolecular interactions (Mohan et al., 2009). Although it has been thought that affinity maturation (that coincides with decreased polyspecificity) results in loss of conformational flexibility and thereby higher specificity, a systematic computational study could not confirm such loss of flexibility (Regenmortel, 2014; Jeliazkov et al., 2018).

It is important to consider that antibody properties like polyspecificity also depend on external factors, hence the specific biochemical and physical environment used in experimental assays can also influence the results and comparability between studies. In this regard, polyspecificity has been shown to be dependent on chemical conditions and not the antibody inherently, i.e. antibodies polyspecific in conventional buffers lost their polyspecificity in serum (Chu et al., 2008). As another example, so-called cryptic polyspecificity can be acquired after exposure to protein-modifying conditions, such as those at sites of inflammation, and common mechanisms depending on the particular modifying condition have been observed (Dimitrov et al., 2010). In this context, a cryptic polyspecific antibody has been shown to acquire binding ability to distinct epitopes on gp120 after exposure to heme (Dimitrov et al., 2014). Furthermore, temperature can affect the properties of antibodies. Somatic hypermutation inversely correlates with promiscuity and self-association, but positively correlates with thermodynamic stability (Lecerf et al., 2019). Interestingly, lowering the temperature decreased the specificity of a mature, specific antibody but did not affect it in case of the polyspecific antibody (Oppezzo et al., 2004). In addition to differences in thermal stability, polyreactive antibodies were shown to be cleared from the circulation faster than monoreactive antibodies and are more likely to be deposited in organs, mainly in the liver (Sigounas et al., 1994). Shehata and others have also confirmed that clearance of human antibodies correlated with their degree of polyspecificity as determined by other assays (Shehata et al., 2019).

A potential factor contributing to polyspecificity could be V gene usage, however, in B1 lymphocytes that are known to be more polyspecific, the V gene usage was not different in polyreactive antibodies from the general V gene distribution (Bhat et al., 1997). This was also shown to be true for the whole antibody repertoire (not limited to B1 cells), and instead glycosylation was suggested as a potential factor explaining polyreactivity (Fernández et al., 1997a; 1997b). Another interesting aspect is the 3D structure of antibody paratopes in the context of polyspecificity however, current methods for structural modeling are computationally expensive and therefore usually not applicable to large data sets.

Nevertheless, recent advances in machine learning have greatly improved opportunities to predict features correlating with desired and undesired properties of antibodies [reviewed in (Makowski et al., 2023a)]. For example, in an exciting recent application of machine learning, scientists mutated sites in a clinical-stage antibody followed by sorting for high *versus* low specificity binding. Deep learning models trained on these data enabled identification of co-optimal levels of specificity and target antigen affinity (Makowski et al., 2022). In a following study, interpretable machine learning leveraging structural features of clinical-stage antibodies was implemented to improve affinity and specificity of several clinical stage antibodies (Makowski et al., 2023b). Such novel methods may accelerate development of therapeutic antibodies in the future.

In conclusion, here we provide a new conceptual framework and method for predicting polyspecificity of antibodies based on the sequence of their heavy chain variable regions, which may in the future be exploited to improve developability, specificity and safety of therapeutic antibodies.

## Data Availability

The original data presented in this study are publicly available at the following accession number and link: PRJNA1052988 on NCBI/NIH https://www.ncbi.nlm.nih.gov/bioproject/?term=PRJNA1052988.
