# Supplementary material for: Prediction of polyspecificity from antibody sequence data by machine learning
Source: Front Bioinform. 2024 Apr 8;3:1286883. doi: 10.3389/fbinf.2023.1286883 (PMC11033685; doi:10.3389/fbinf.2023.1286883)
Supplement: Supplementary file 1 [file Image1.PDF]

Supplementary Figure 1

A)

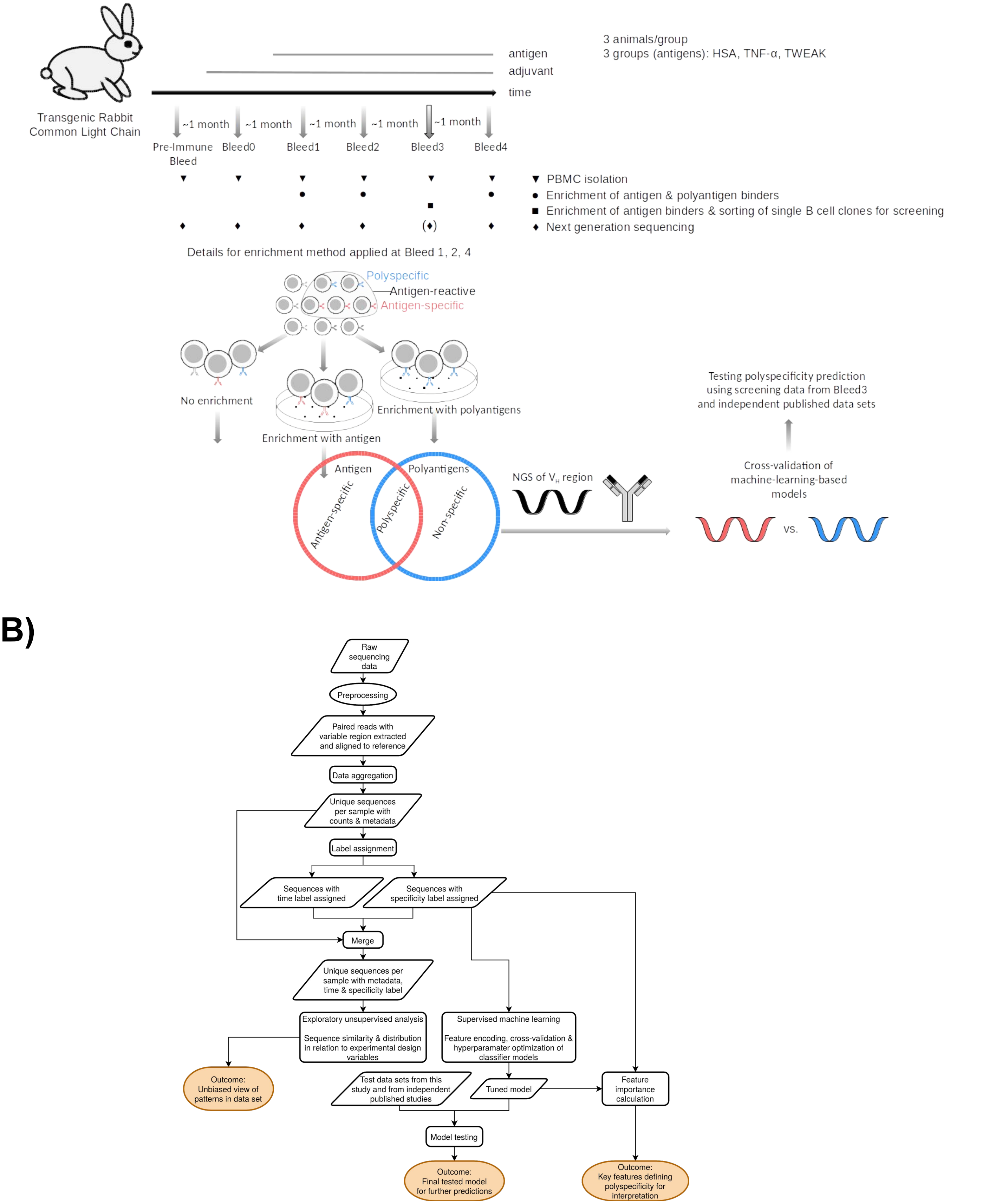

Supplementary Figure 1. Schematic representation of the experimental design and computational procedure for polyspecificity prediction. A) Illustration of the experimental process including immunization, sampling time points, enrichment using antigens and polyantigens, next-generation sequencing of the region coding for the variable region of the heavy chain. B) Illustration of the computational steps including preprocessing, data aggregation, data preparation, machine-learning-based modeling and performance estimation.

Supplementary Figure 2

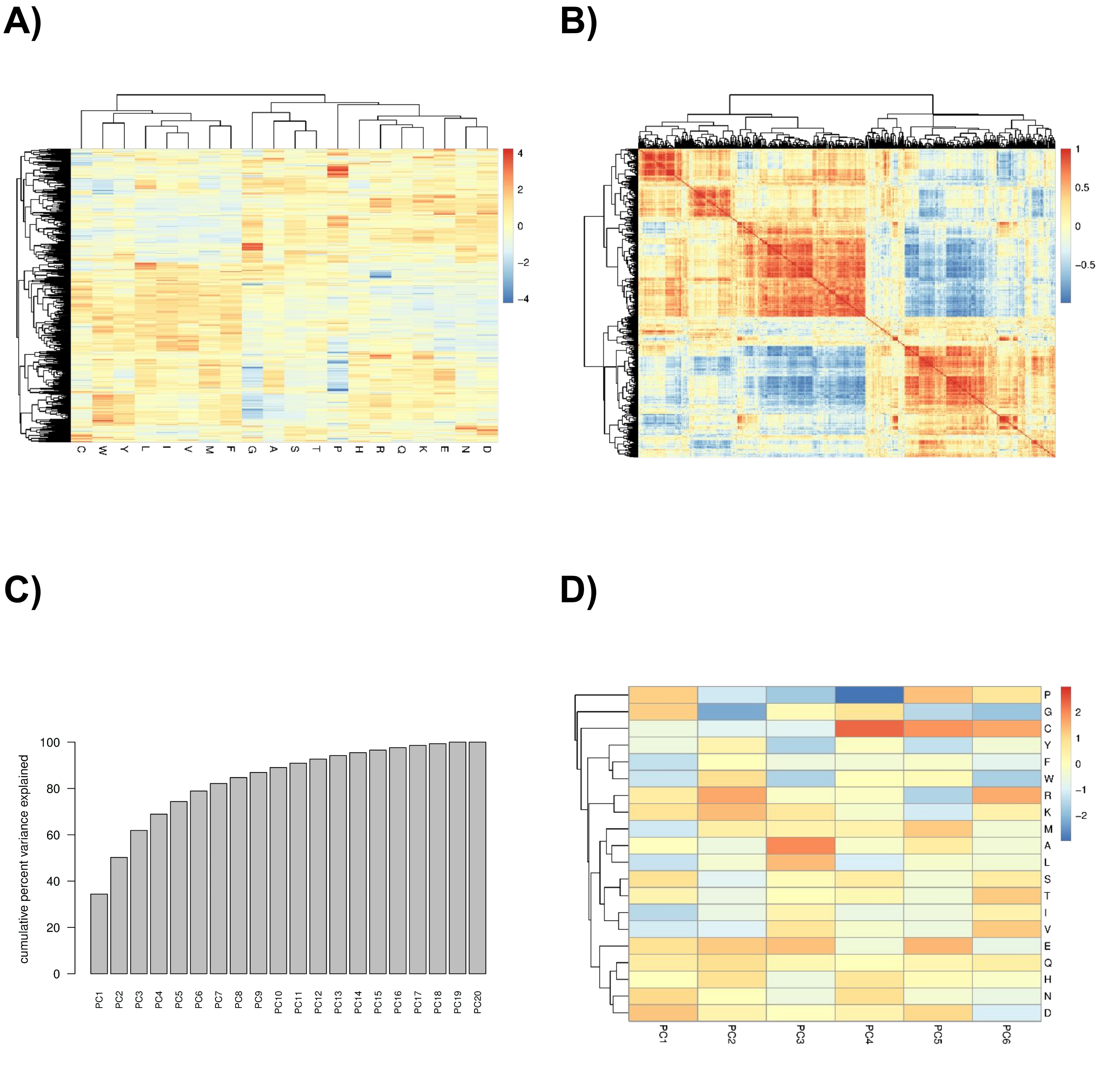

Supplementary Figure 2. PCA-based feature encoding using physico-chemical properties of amino acids. A) Physico-chemical properties (rows) of amino acids (columns) used for feature encoding. B) Spearman correlation between physico-chemical features. C) Percent cumulative variance explained by the principal components. D) Values on the first six principal components corresponding to amino acids.

Supplementary Figure 3

A)

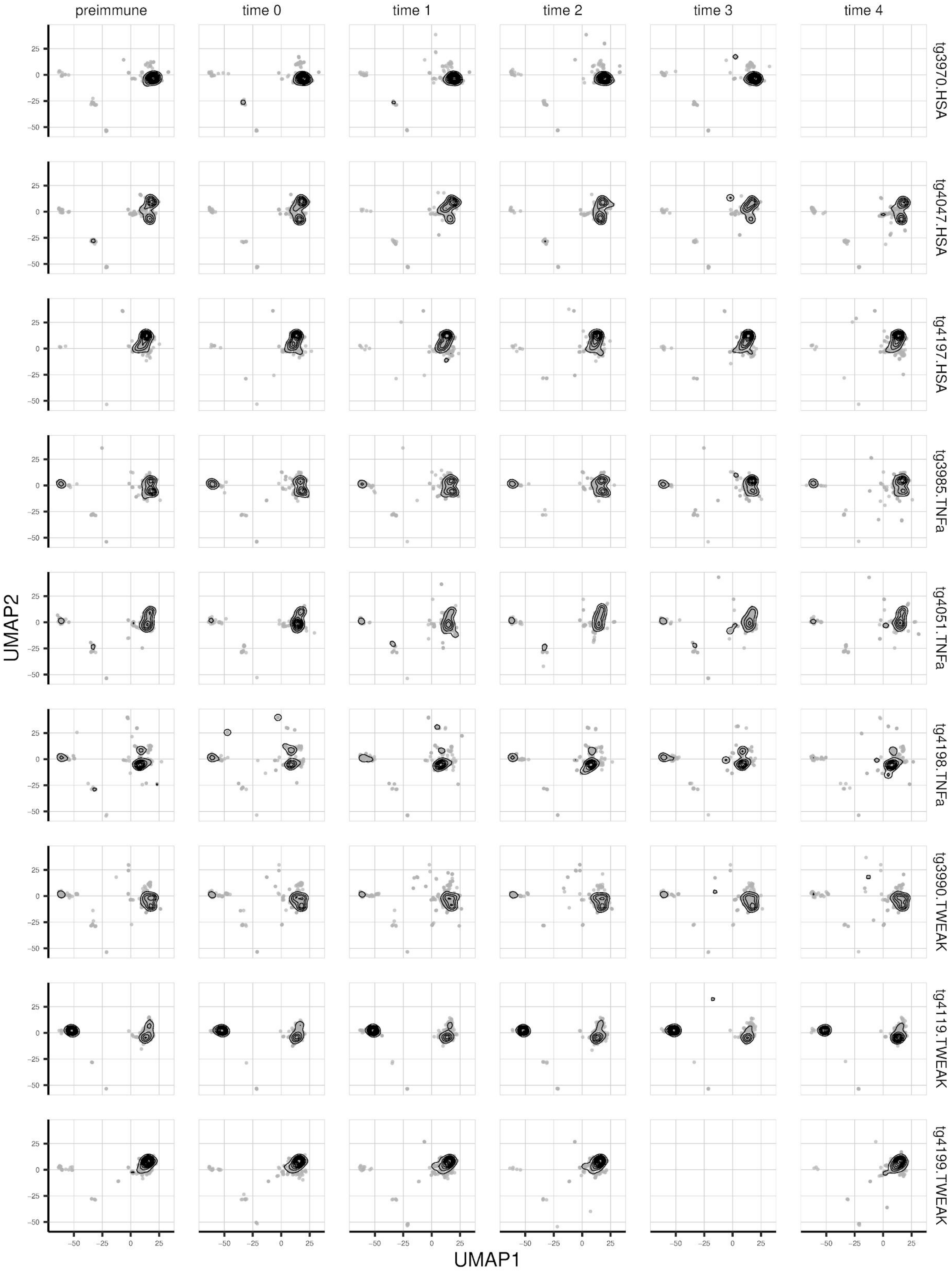

Supplementary Figure 3

B)

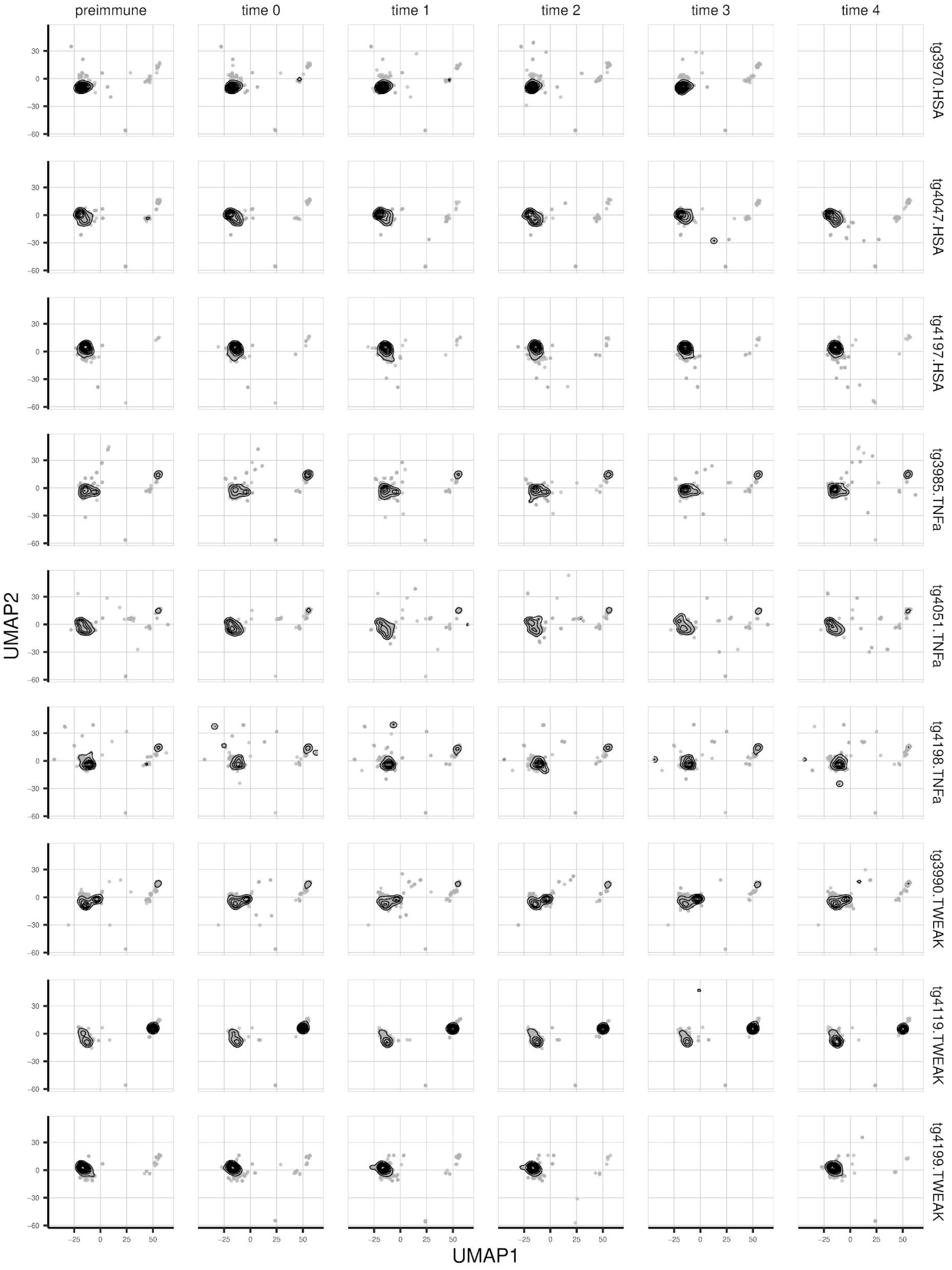

Supplementary Figure 3

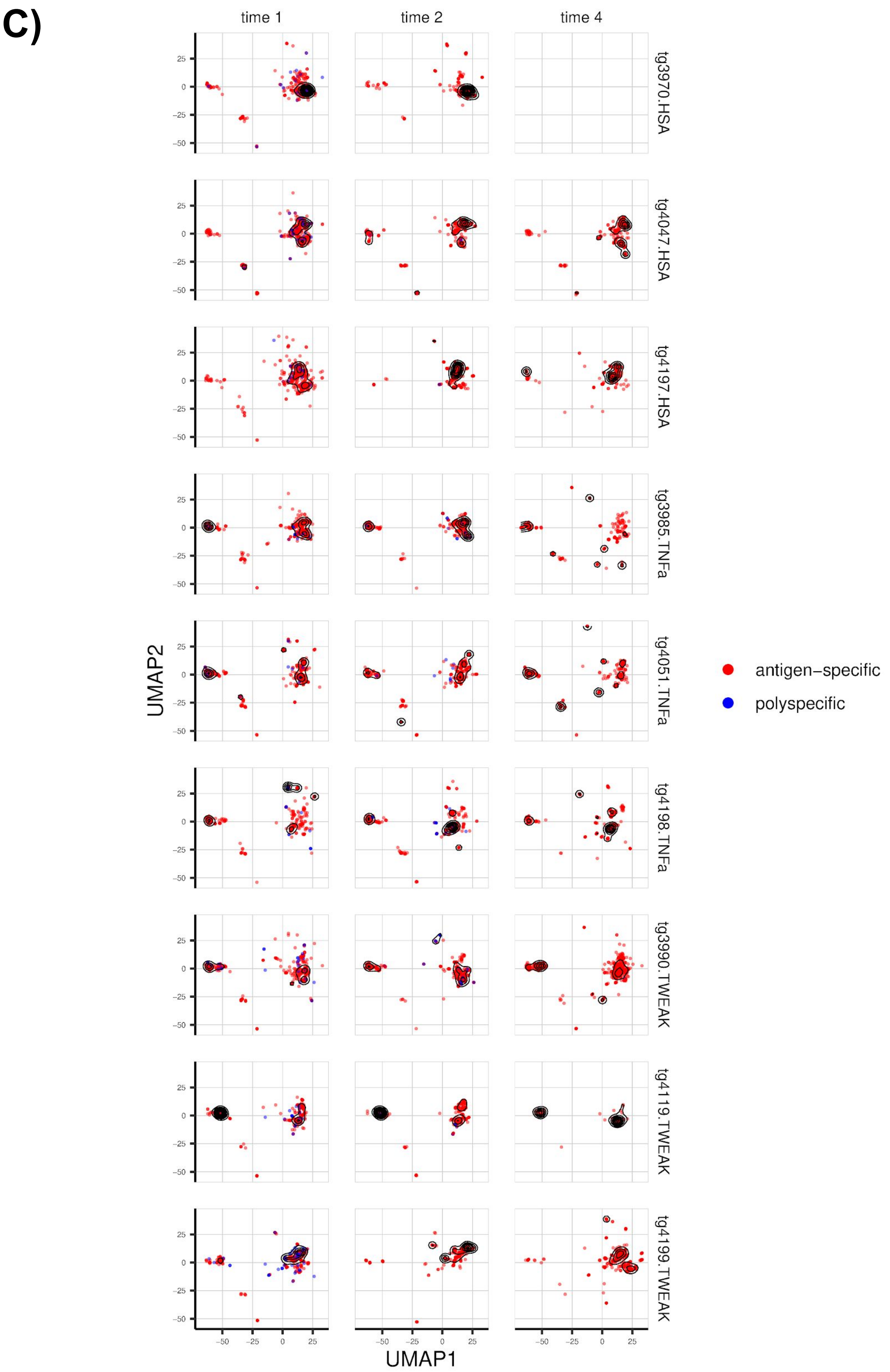

Supplementary Figure 3

D)

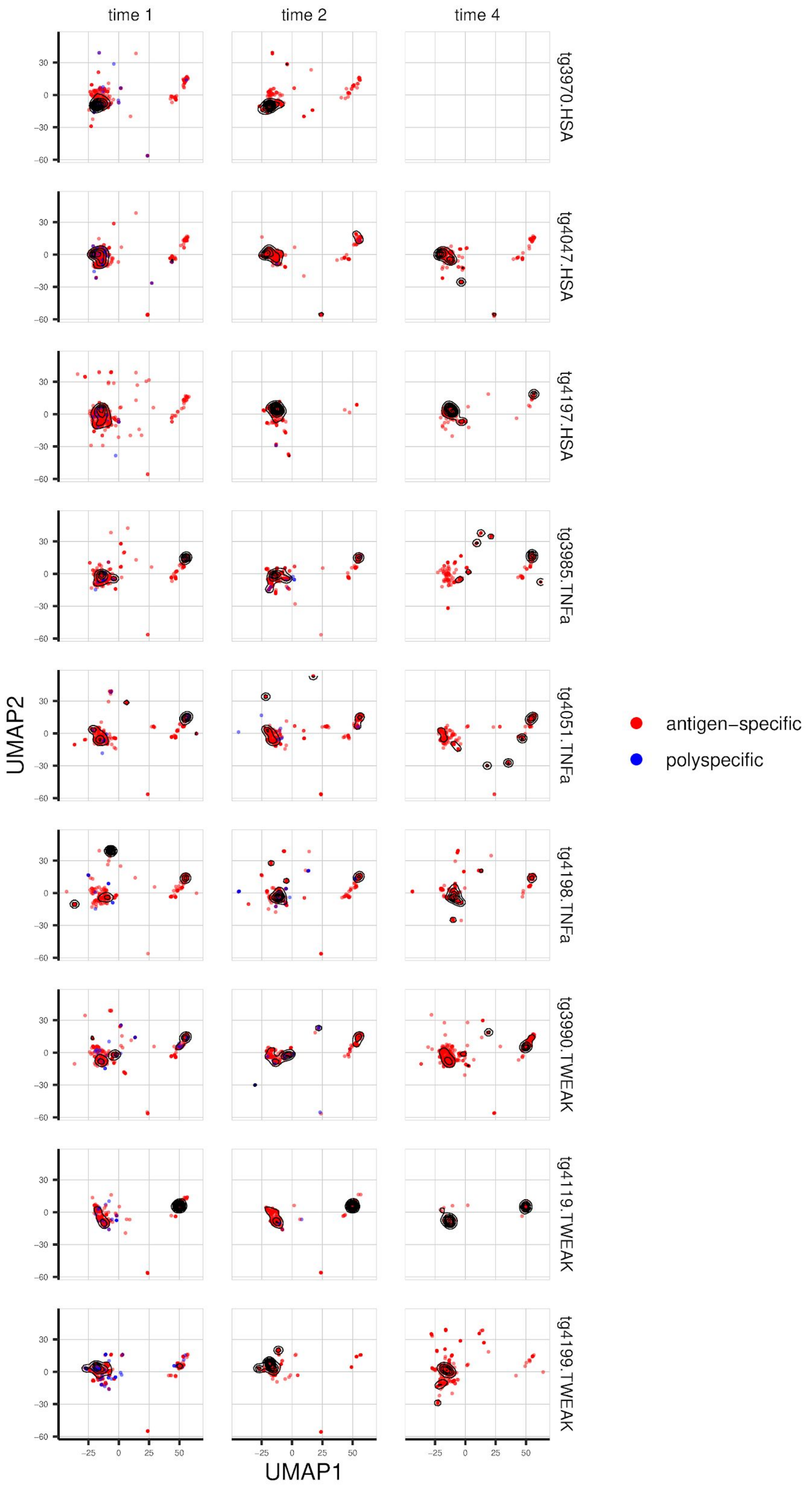

Supplementary Figure 3. Similarity and distribution of antibody clones with respect to experimental design parameters. A) UMAP projection using randomly sampled antibody clone sequences encoded to integers as input and Hamming distance as distance metric. B) UMAP projection using the same antibody clone sequences as in (A) encoded to PCA-based physico-chemical feature vectors as input and Euclidean distance as distance metric. C) UMAP projection using polyspecific and antigen-specific antibody clone sequences encoded to integers as input and Hamming distance as distance metric. D) UMAP projection using the same antibody clone sequences as in (C) encoded to PCA-based physico-chemical feature vectors as input and Euclidean distance as distance metric.

Supplementary Figure 4

A)

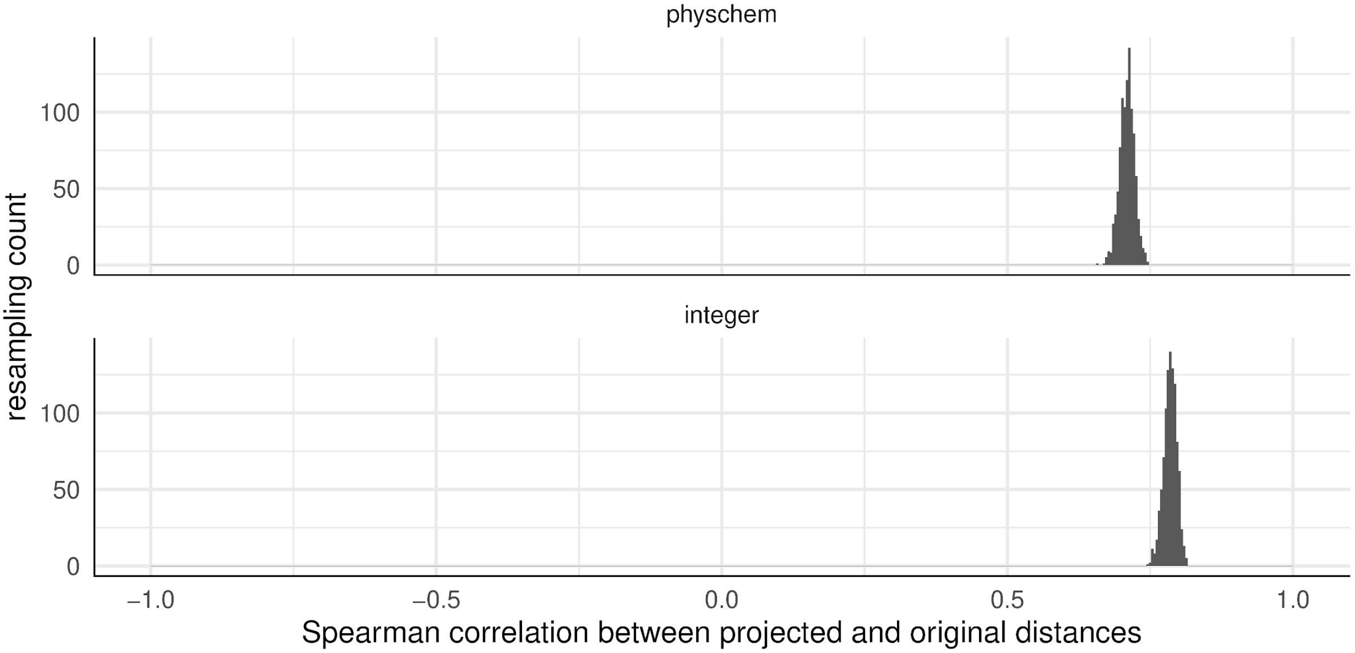

B)

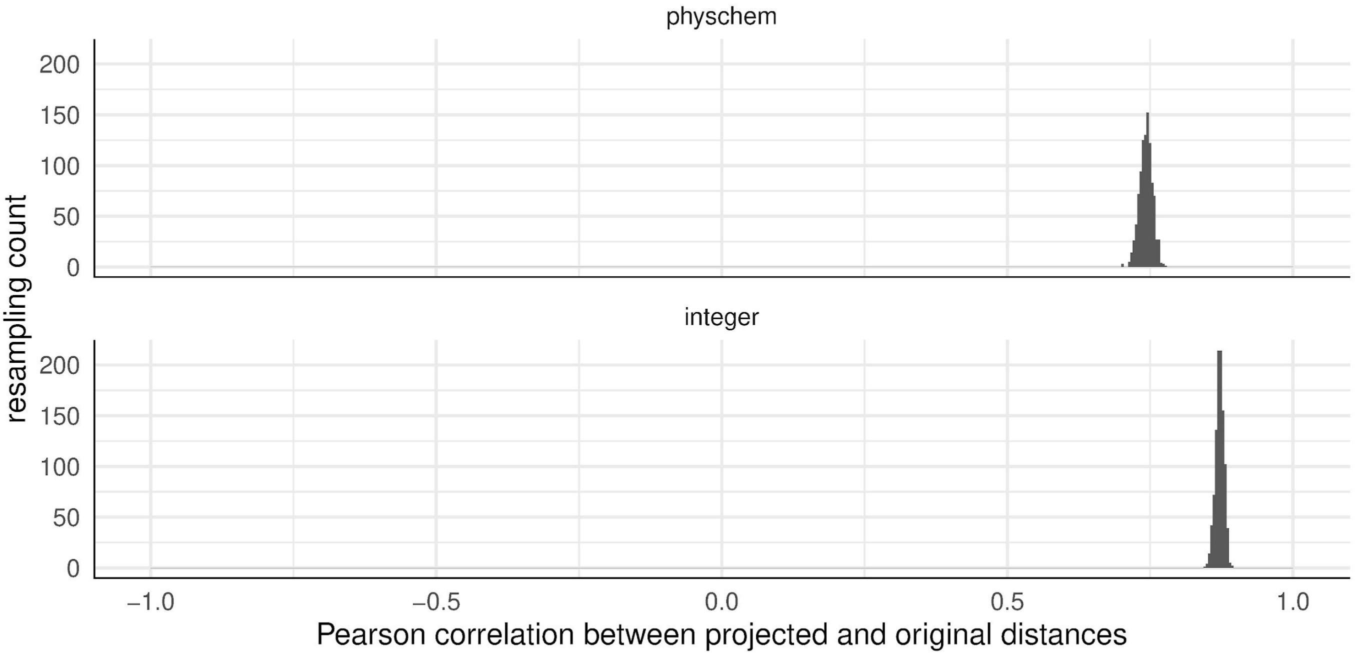

Supplementary Figure 4. Estimating the performance of UMAP-based dimensionality reduction by quantifying the preservation of distances. A) Spearman correlation is shown between the pairwise distances in the initial (unprojected) space and in the UMAP-projected space. Upper panel: For UMAP, sequences encoded using PCA-based physico-chemical feature vectors were used with Euclidean distance as distance metric, and the pairwise distances in the unprojected and projected space are both calculated using Euclidean distance. Lower panel: For UMAP, sequences encoded using integer feature vectors resulting from the one-to-one mapping of amino acids to integers were used with Hamming distance as distance metric. The pairwise distances in the unprojected space are calculated using the Hamming distance (number of mismatches between aligned sequence-pairs) and the pairwise distances in the UMAP-projected space are calculated using the Euclidean distance. B) Same as in (A) but using Pearson correlation. For the calculations in (A) and (B) a subsampling approach was used whereby 1000 sequences were sampled randomly for a given calculation and this was repeated 1000 times, resulting in the distribution shown.

Supplementary Figure 5

A)

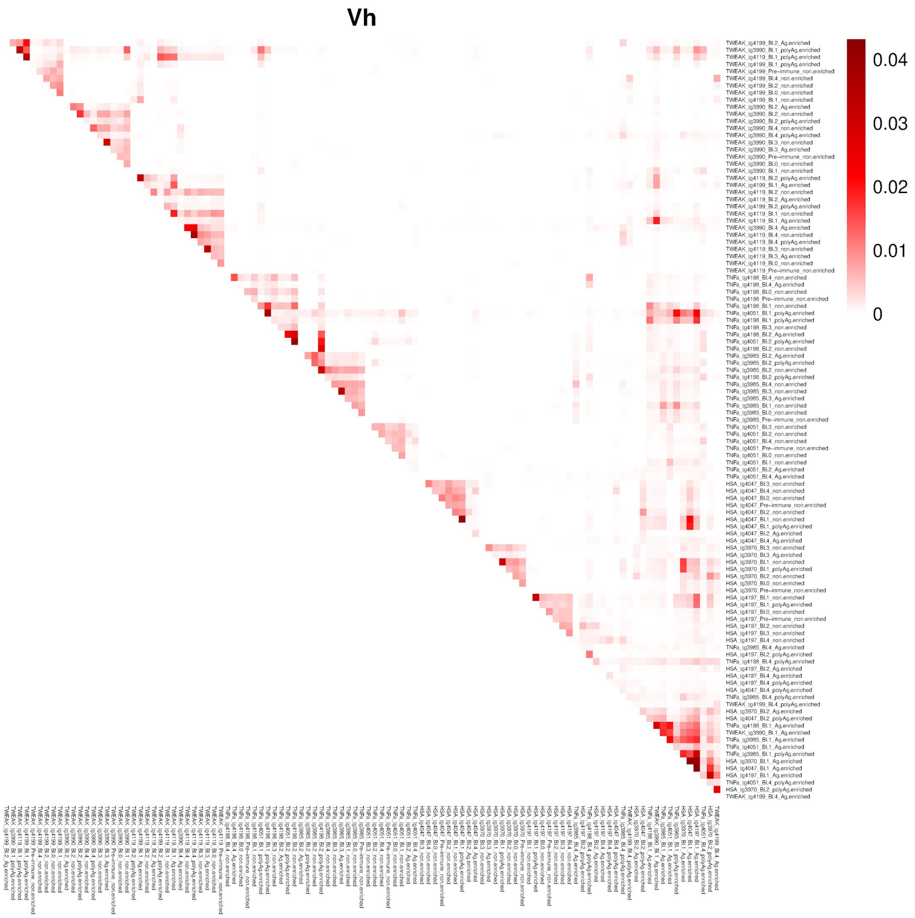

B)

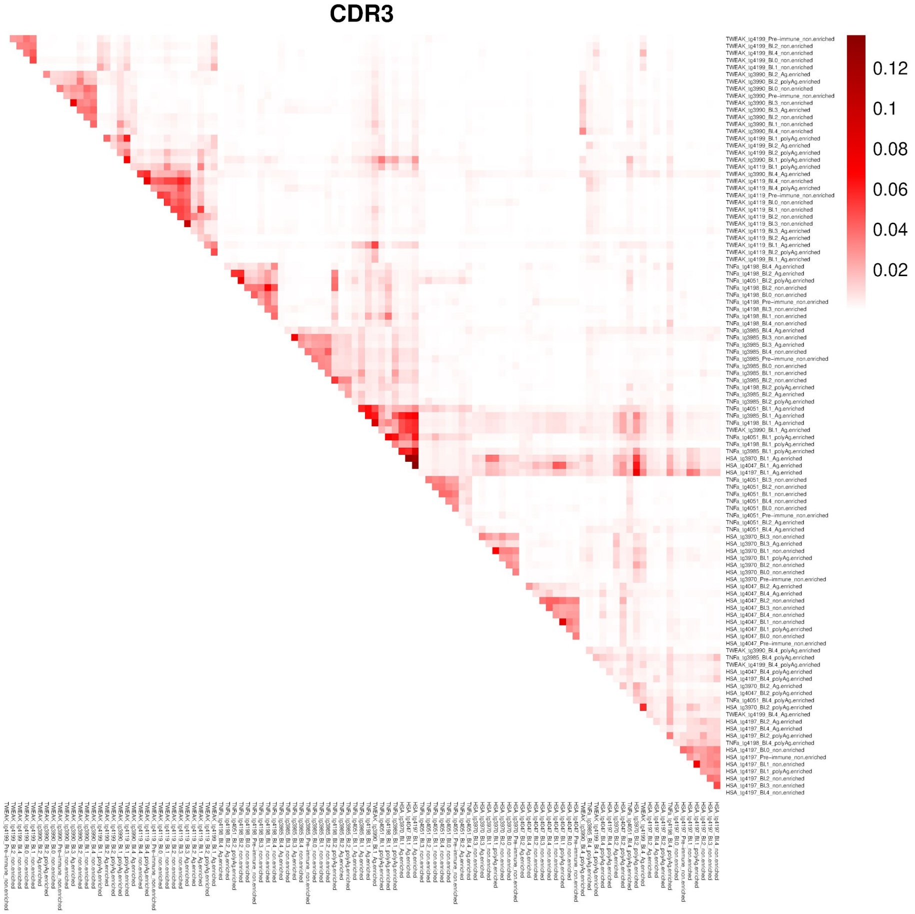

Supplementary Figure 5. Jaccard index between sets of unique sequences that correspond to unique experimental design parameter sets. A) Jaccard index is shown using the VH sequences as set elements. B) Jaccard index is shown using the CDR3 sequences as set elements.

Supplementary Figure 6

A)

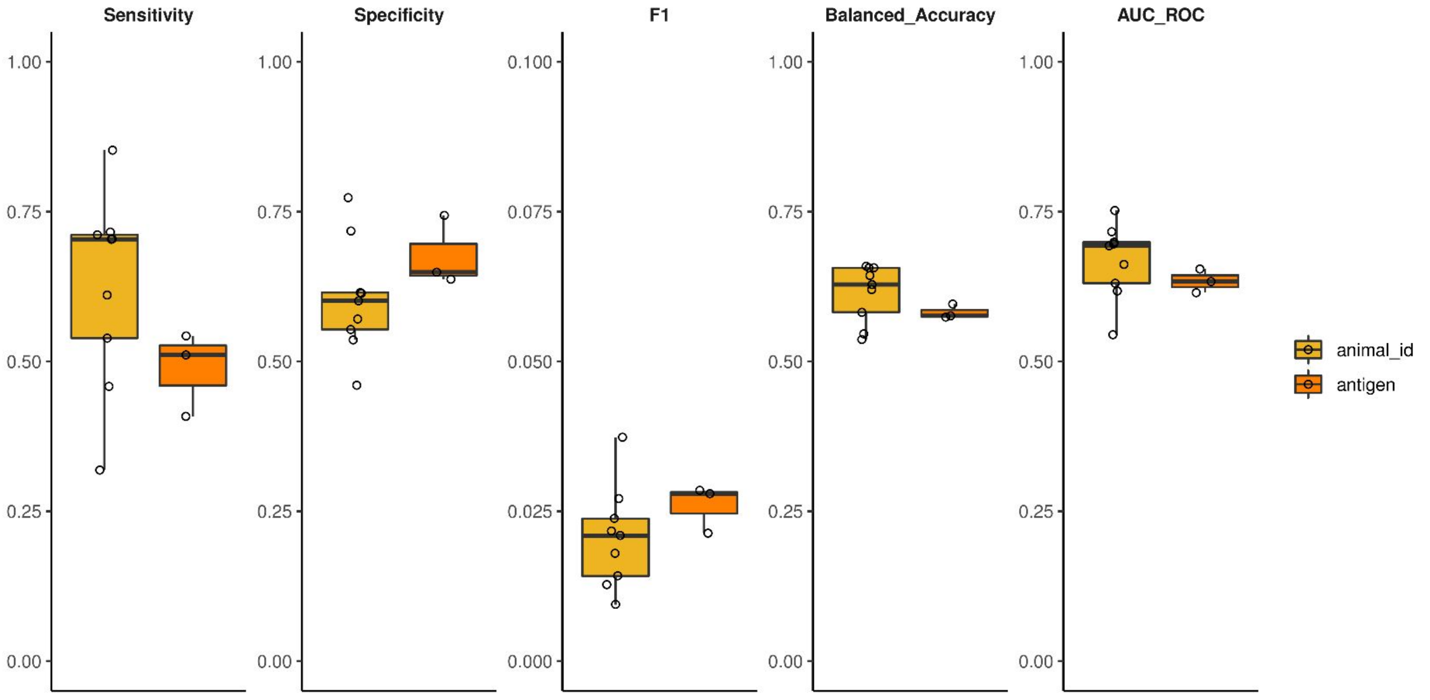

B)

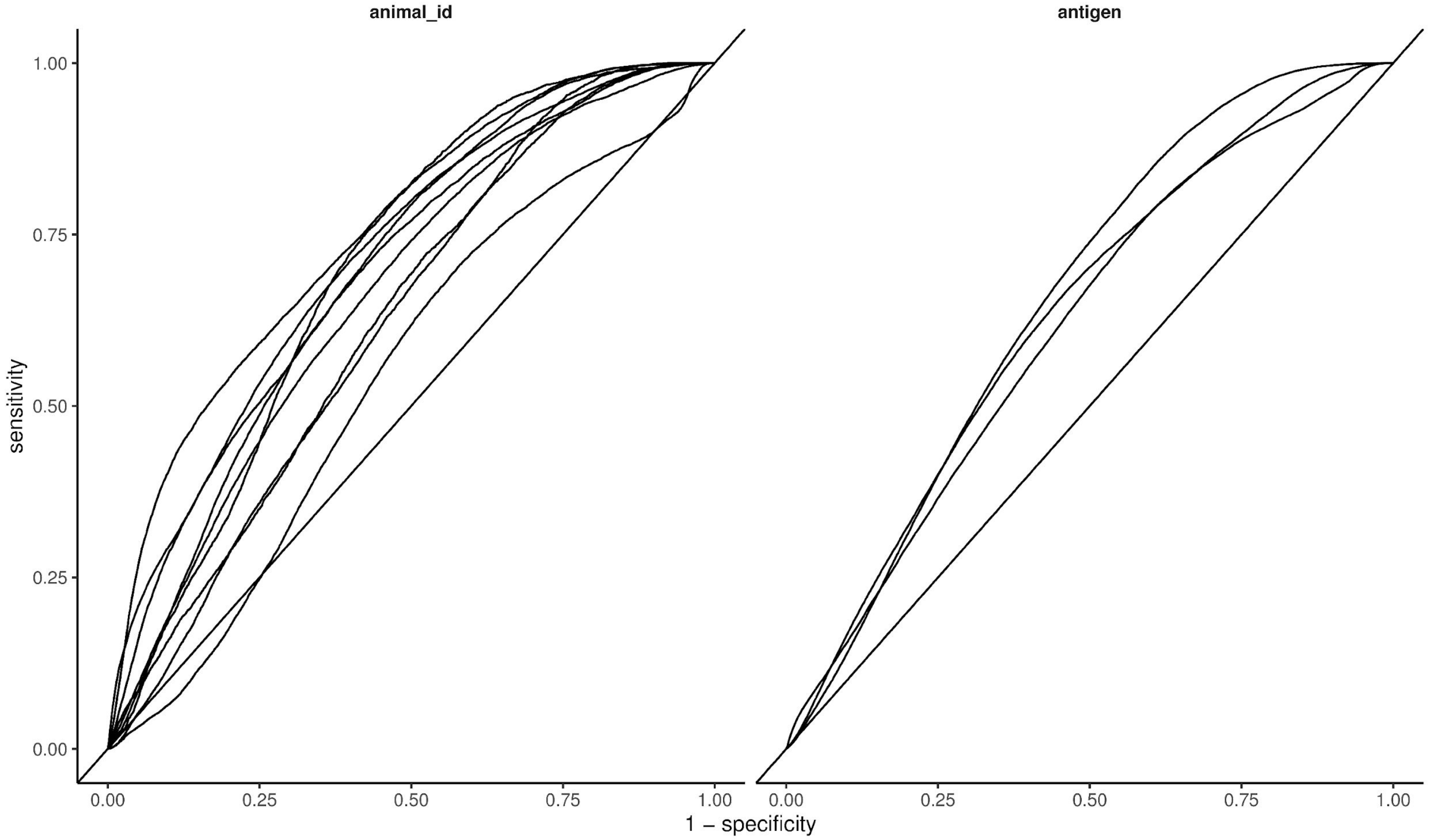

Supplementary Figure 6. Performance estimation of models predicting antibody polyspecificity using antigen-blocked and animal-blocked cross-validation. A) Performance metrics are shown as a summary of a 10-fold cross-validation with animal-based or antigen-based blocked data splitting. B) ROC curves resulting from the same setup and data as in A).

Supplementary Figure 7

A)

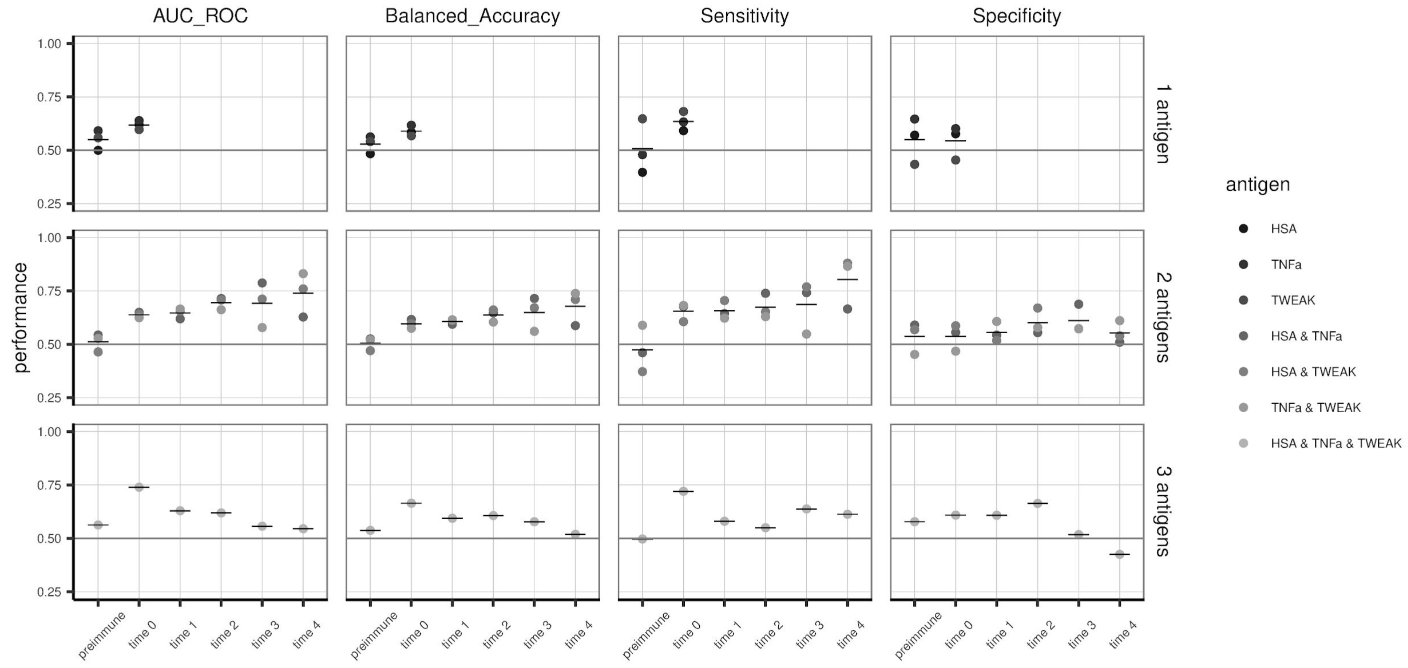

B)

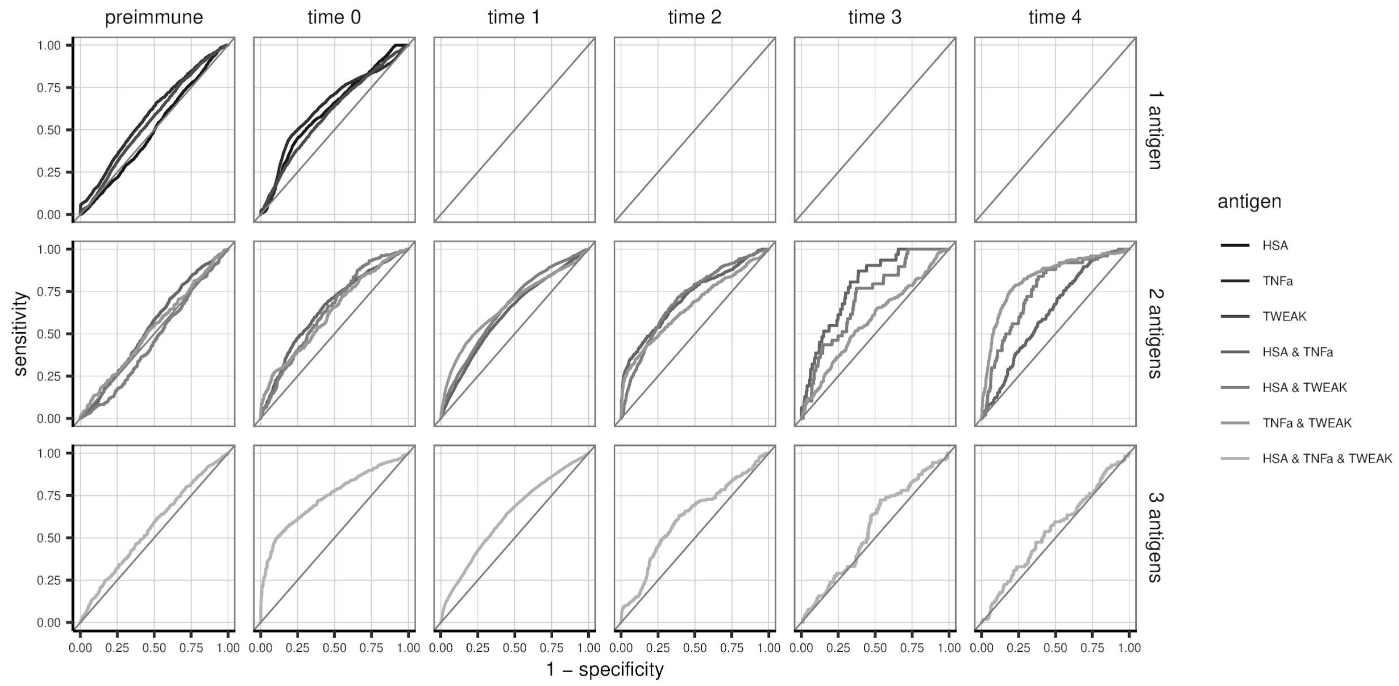

Supplementary Figure 7. Performance estimation of the model that predicts antibody polyspecificity using the NGS-derived VH sequences that were excluded from the training. A) Different performance metrics are quantified and shown using the sequence sets from the NGS data that were excluded from the model training because they were (i) observed before immunization with the antigen (i.e. first observed at pre-immune or bleed 0 time point) and/or (ii) shared between any of the immunization groups. The class labels ("polyspecific" or "antigen-specific") for the clones (i) were assigned based on the enrichments performed at subsequent time points (since experimental enrichment with polyantigens or antigen was only performed after immunization with the antigen, see Supplementary Figure 1A). B) Same as in (A) but showing ROC curves.

Supplementary Figure 8

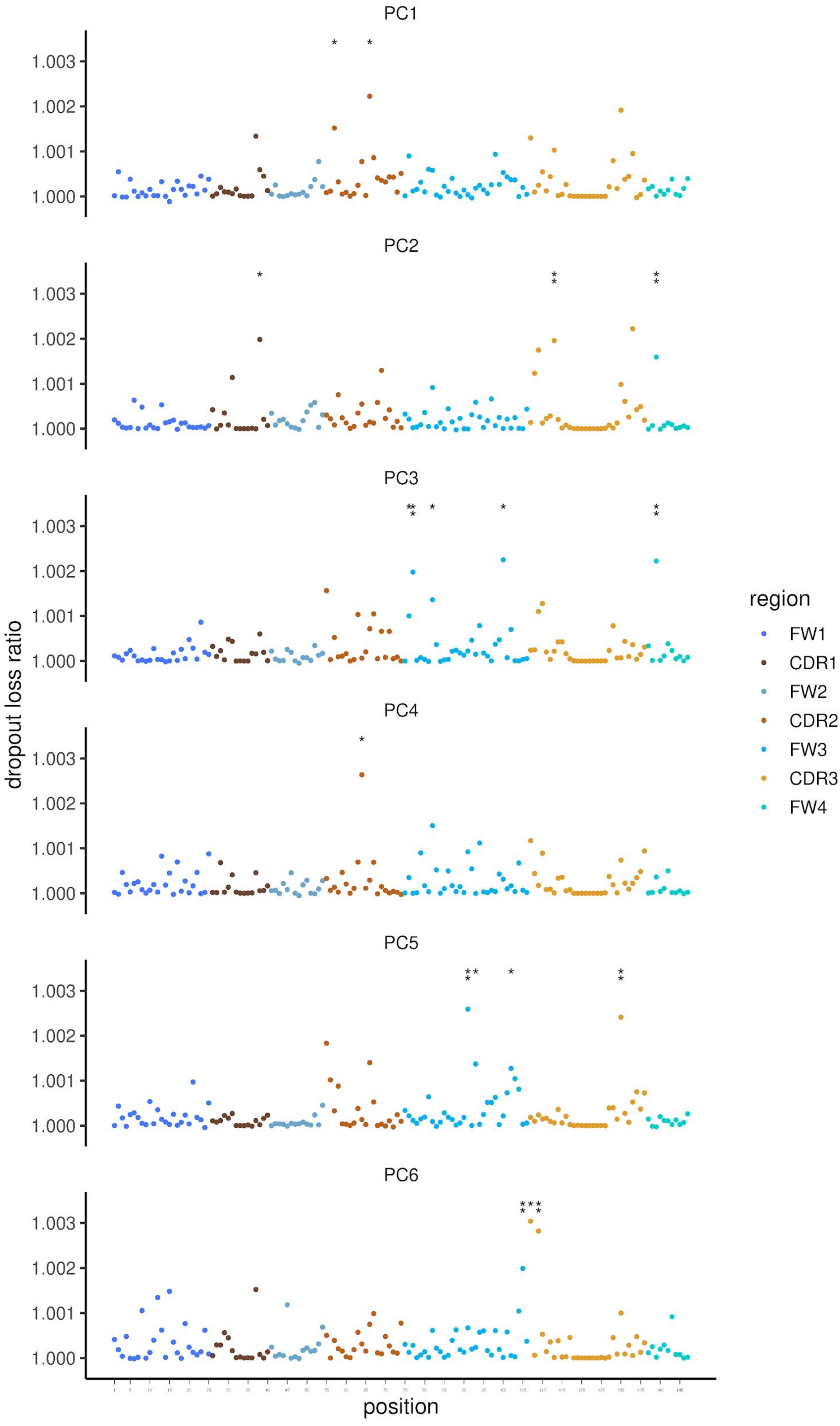

Supplementary Figure 8. Feature importance for polyspecificity prediction. Importance values corresponding to the features used for classification are shown along the positions in the VH region. Feature importance is shown as the loss ratio (dropout loss ratio) calculated using predictions from the model with permuted and unpermuted values for each feature. Importance for polyspecificity prediction is indicated by values above 1. Subplots are arranged by the principal component corresponding to each feature. Dots represent the mean values across resampling rounds and stars indicate statistical significance based on FDR (* indicates FDR < 0.1; ** indicates FDR < 0.05).

Supplementary Figure 9

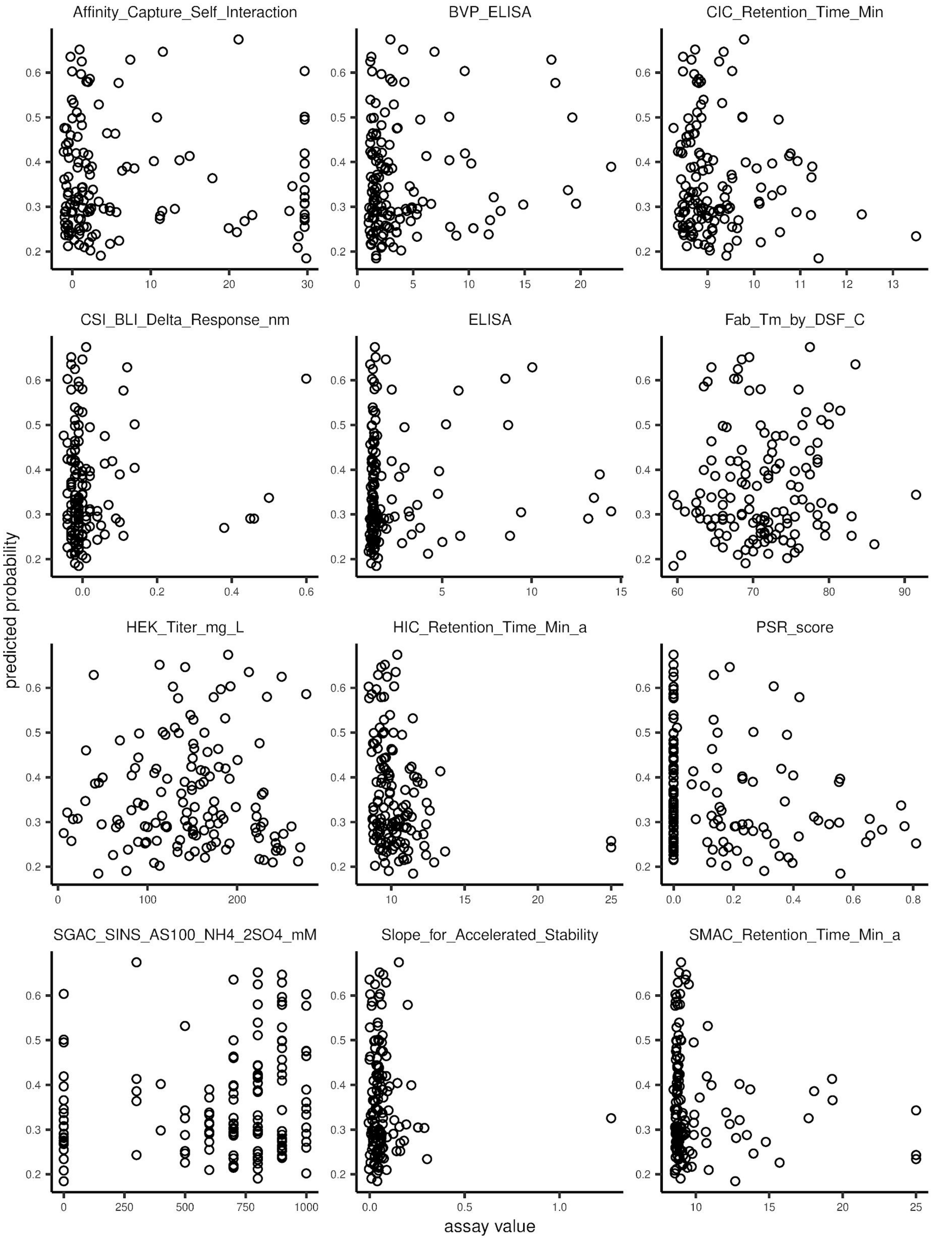

Supplementary Figure 9. Association between predicted polyspecificity by the model and physico-chemical properties using the data set from Jain et al. 2017. Scatter plots show the measured values from the given assay and the predicted probability of polyspecificity for the antibodies from the published data set.

Supplementary Figure 10

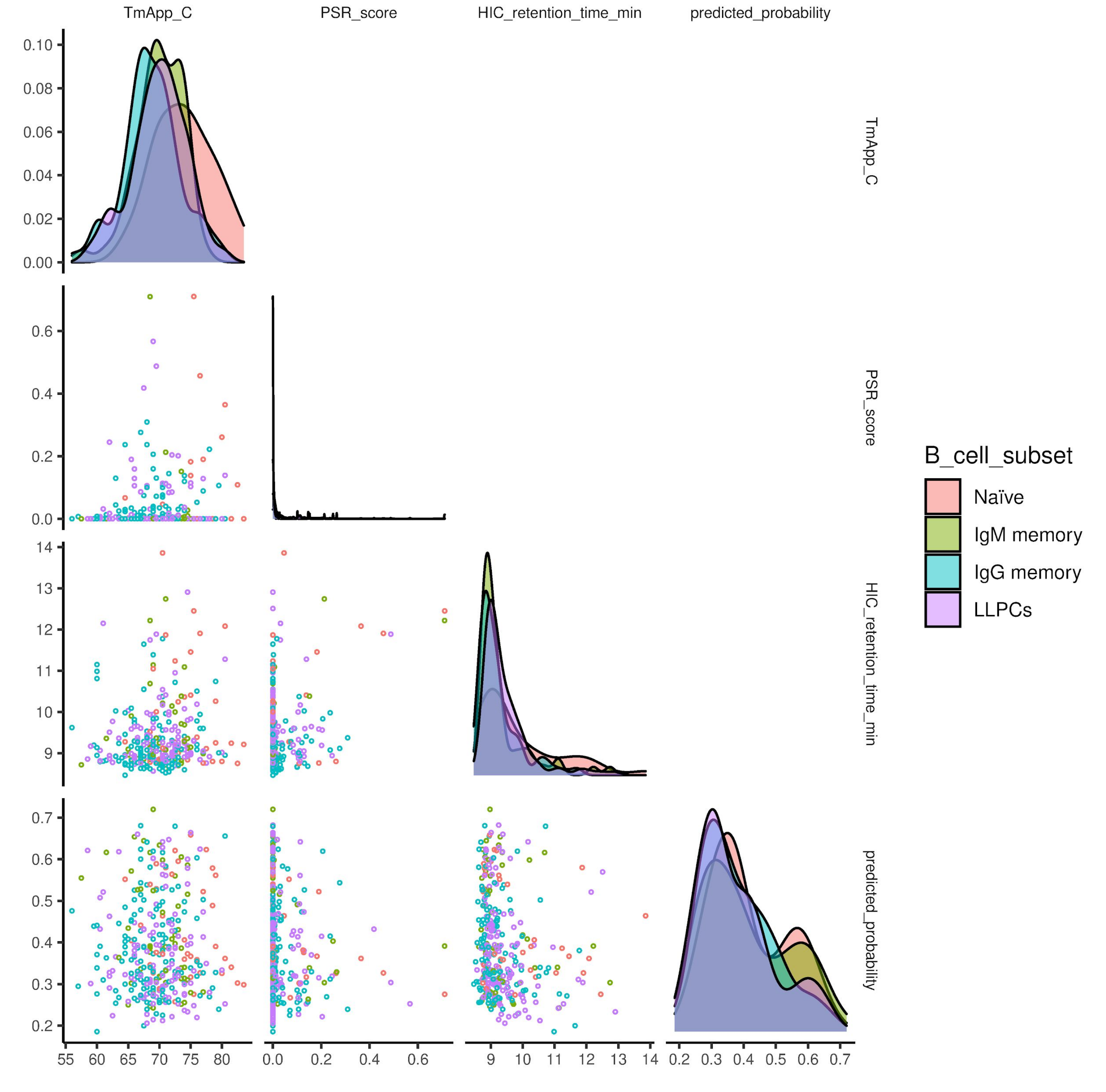

Supplementary Figure 10. Association between predicted polyspecificity by the model and physico-chemical properties using the data set from Shehata et al. 2019. Scatter plots show the measured values from the given assay and the predicted probability of polyspecificity for the antibodies from the published data set. Density plots on the diagonal show the distribution of the given assay measurements. Colors indicate different B cell subset according to maturation.

Supplementary Figure 11

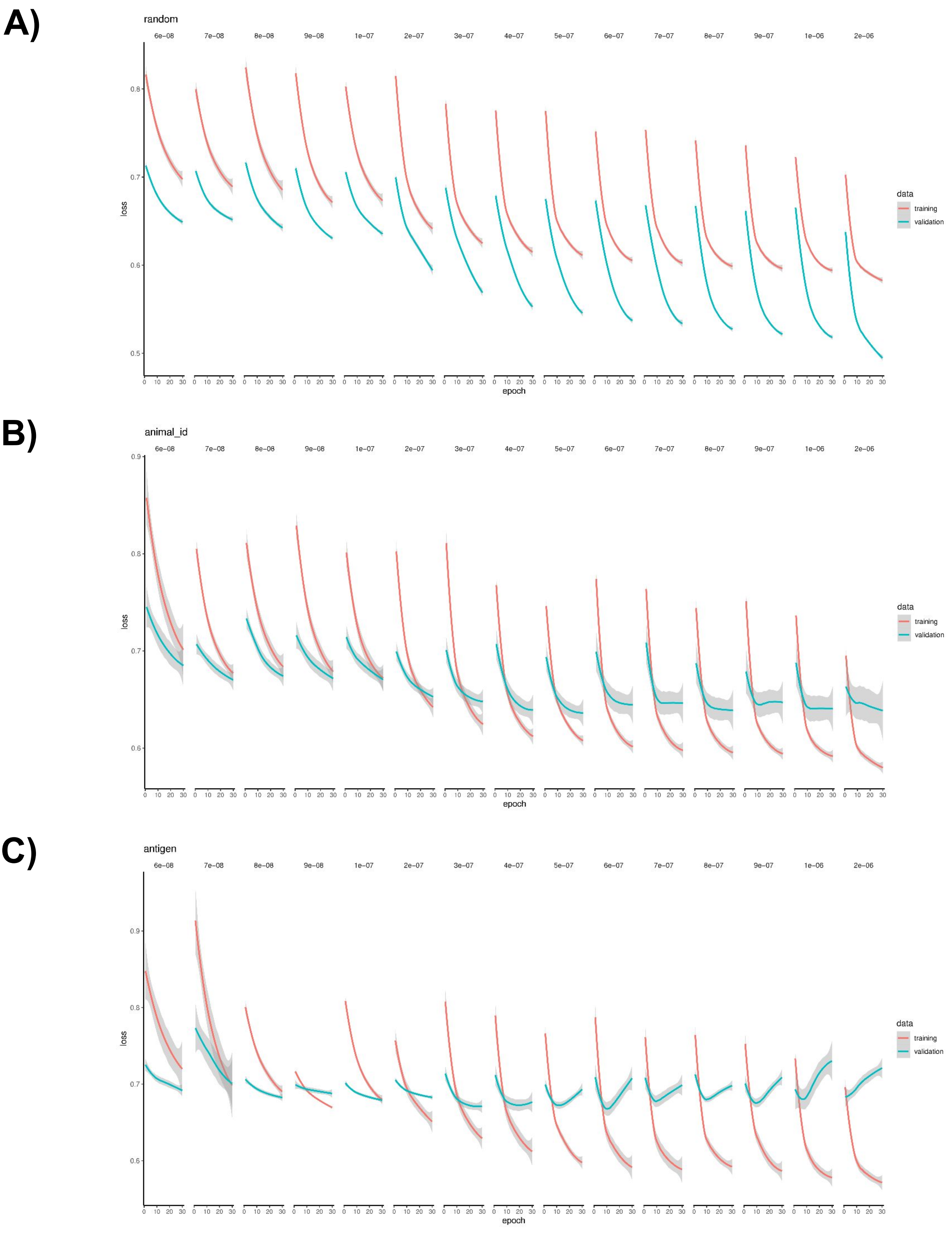

Supplementary Figure 11. Training and validation loss across epochs with different learning rates and data splitting for cross-validation. Mean loss values (smoothed) of the cross-validation rounds are shown for different number of training epochs. Colors indicate the loss value for the training or validation data partition. Subplots indicate different learning rate settings. A) 10-fold cross-validation using random splitting of the data. B) Blocked cross-validation splitting the data based on the animals from which the sequences were identified. C) Blocked cross-validation splitting the data based on the antigens (immunization groups) from which the sequences were identified.
